# ﻿Review of the spider genus *Solenysa* Simon, 1894 in Western Japan and Central Ryukyu with the description of three new species (Araneae, Linyphiidae)

**DOI:** 10.3897/zookeys.1232.142629

**Published:** 2025-03-17

**Authors:** Francesco Ballarin, Hauchuan Liao, Zento Touyama, Katsuyuki Eguchi

**Affiliations:** 1 Systematic Zoology Laboratory, Department of Biological Sciences, Tokyo Metropolitan University, 1-1 Minami-Osawa, Hachioji-shi, 192-0397, Tokyo, Japan Tokyo Metropolitan University Tokyo Japan; 2 Department of Zoology, Museo di Storia Naturale of Verona, Lungadige Porta Vittoria, 9, I-37129 Verona, Italy Museo di Storia Naturale of Verona Verona Italy; 3 Department of Entomology, National Chung Hsing University, No.145 Xingda Rd., South Dist., Taichung City, 402202, Taiwan National Chung Hsing University Taichung City Taiwan; 4 Laboratory of Entomology, Department of Bioresource Development, Faculty of Agriculture, Tokyo University of Agriculture, 1737 Funako, Atsugi-shi, 243-0034, Kanagawa, Japan Tokyo University of Agriculture Kanagawa Japan

**Keywords:** Amami-Ōshima, endemic species, island biogeography, Kyushu, Okinawa, Ryukyu Archipelago, Yambaru National Park

## Abstract

Three new species of the genus *Solenysa* Simon, 1894 are recorded and described from Western Japan, all based on both sexes. Of these, two species are endemic to the Ryukyu Archipelago: *S.shimatchu* Ballarin & Eguchi, **sp. nov.** from Amami-Ōshima Island, and *S.yambaruensis* Ballarin & Eguchi, **sp. nov.** from Okinawa Honto and Kume-jima Islands. These species represent the first record of the genus *Solenysa* for the Central Ryukyus and belong to a newly defined species group. Another new species, *S.bilamellata* Ballarin & Eguchi, **sp. nov.** is recorded from eastern Kyushu in mainland Japan and belongs to the *mellotteei* group. The phylogenetic positions of the new species are discussed based on morphological and molecular data. New records, remarks, and photos of genitalia of other poorly known *Solenysa* species from surrounding areas (Taiwan, Kyushu, Shikoku, and western Honshu) are provided to facilitate future identifications.

## ﻿Introduction

*Solenysa* Simon, 1894 is a small genus of tiny, sheet-web weavers spiders belonging to the family Linyphiidae Blackwall, 1859. These spiders are easily recognizable from other linyphiid genera by having distinctive somatic features, such as a reddish-colored prosoma, a carapace with a microsculpture of rounded pits on the surface, and a raised cephalic area. Additionally, *Solenysa* species show uniquely shaped genitalia, including a mobile epigyne hanging from the distal end of a long solenoid (Tu and Hormiga 2011). *Solenysa* was originally erected by Simon (1894) based on the type species *Solenysamellotteei* Simon, 1894 from Japan. The genus currently comprises 15 species divided into four species groups, all distributed in Eastern Asia, including mainland China (six species), Taiwan (three species), Korea (one species), and mainland Japan (six species) (Wang et al. 2015; WSC 2024).

The taxonomy, phylogeny, and diversification of *Solenysa* have been studied in relatively good detail in previous research (Tu and Li 2006; Tu and Hormiga 2011; Wang et al. 2015; Tian et al. 2022). However, its systematic position within the subfamilies of Linyphiidae remains controversial (Wang et al. 2015). A recent study has proposed an ancient origin for this genus, suggesting *Solenysa* as a possible Cretaceous relict with an old evolutionary history and a late Cenozoic diversification (Tian et al. 2022). This, together with the possible need for specific microhabitats (e.g., humid forest leaf litter), may explain the relatively high level of endemism observed in this genus. Such a combination of features makes *Solenysa* a potentially interesting model taxon for future studies on the historical biogeography of Eastern Asia.

In Japan, six species of *Solenysa* are known to occur, all belonging to the *S.mellotteei* group sensu Tu and Hormiga (2011): *Solenysamacrodonta* Wang, Ono & Tu, 2015; *S.mellotteei* Simon, 1894; *S.ogatai* Ono, 2011; *S.partibilis* Tu, Ono & Li, 2007; *S.reflexilis* Tu, Ono & Li, 2007; and *S.trunciformis* Wang, Ono & Tu, 2015 (Tanikawa 2024; WSC 2024). The classification of the Japanese *Solenysa* species has been revised in detail by Tu et al. (2007) and Wang et al. (2015). However, the number of documented records remains relatively low and scattered across mainland Japan (Wang et al. 2015; Shinkai et al. 2024). Currently, no species of *Solenysa* have been recorded on the numerous islands forming the Ryukyu Archipelago except for a very recent finding of *S.reflexilis* on Yakushima (Shinkai and Tanikawa 2023), one of the archipelago’s northernmost islands located approximately 70 km far from the southern coast of Kyushu. This absence of records in most of the Ryukyus is surprising given the archipelago’s central position within the known distributional range of *Solenysa*, highlighting a significant gap in our geographic knowledge of the genus.

During recent field surveys in Taiwan, the Ryukyus, and western Japan, we conducted extensive forest leaf litter sifting, collecting numerous linyphiids species, including several individuals of *Solenysa*. A detailed morphological comparison of these specimens with other congeners revealed the presence of three undescribed species, two from the Ryukyus and one from mainland Kyushu, Japan.

In this study, we aim to report the new records for the Central Ryukyus and describe the new species based on both sexes. To aid future identifications, we provide detailed illustrations of their diagnostic characters, along with images and distribution notes for other poorly known *Solenysa* species endemic to the surrounding areas of the Ryukyus, including Taiwan, Kyushu, Shikoku, and western Honshu. Additionally, we use molecular data to support the validity of the new species and their phylogenetic relationships within *Solenysa*.

## ﻿Material and methods

Specimens were collected by sieving forest leaf litter with an entomological litter reducer and immediately preserved in 99% ethanol for both morphological and molecular analyses. The molecular and morphological studies were conducted in the Laboratory of Systematic Zoology, Department of Biological Sciences, Tokyo Metropolitan University, Japan (**TMU**). Specimens were examined under a Nikon SMZ1270 stereo microscope and a Nikon Optiphot 2 biological microscope. The left male palp and female epigyne were removed from the bodies using a sharp needle to facilitate the observation of diagnostic characters. Internal structures of epigynes were observed by dissecting the epigynes and macerating them in lactic acid for a few hours. Photos were taken using a Canon EOS Kiss X8 digital camera mounted on the same microscopes used for morphological examination. Final images were assembled with Helicon Focus v. 7 image stacking software (https://www.heliconsoft.com) and edited with Adobe Photoshop CC v. 20.0.6 (https://www.photoshop.com/). Lengths of leg segments were measured on the lateral side and are given as follows: total length (femur, patella, tibia, metatarsus, tarsus). All measurements are given in millimeters.

All vouchers used in this study are preserved in the following institutions and collections: the National Museum of Nature and Science, Tsukuba, Japan (**NSMT**); the Museum of Nature and Human Activities, Hyogo, Japan (**MNHAH**); the Museo Civico di Storia Naturale of Verona, Italy (**MSNVR**); the Taiwan Agricultural Research Institute, Taiwan (**TARI**); the Tokushima Prefectural Museum (**TKPM**); and in the personal collection of Francesco Ballarin (FBPC) and Zento Touyama (**ZTPC**).

The following abbreviations are used in the text and figures (after Tu and Hormiga 2011):

Male palp: **AP** anterior protrusion of MTA; **ATA** anterior terminal apophysis; **DSA** distal suprategular apophysis; **E** embolus; **LA** lamella; **LA1** anterior branch of LA; **LA2** median branch of LA; **LA3** posterior branch of LA; **MP** median protrusion of MTA; **MTA** median terminal apophysis; **P** paracymbium; **PBP** probasal cymbial apophysis; **PP** posterior protrusion of MTA; **PTA** posterior terminal apophysis; **PTP** proximal tibial apophysis; **R** radix; **RLP** cymbial retrolateral process; **STT***Solenysa* tegular triangle; **T** tegulum; **VLP** ventral lobe of paracymbium.

Epigyne and vulva: **CD** copulatory duct; **CO** copulatory opening; **DP** dorsal plate; **EC** epigynal collar; **FD** fertilization duct; **LDP** lobe of the dorsal plate; **S** spermatheca; **SL** solenoid; **VP** ventral plate

Other: **TmI** position of trichobothrium on metatarsus I; **TmIV** trichobothrium on metatarsus IV.

### ﻿Molecular analysis

Total genomic DNA was extracted using four legs of each sample using a Chelex-TE-ProK method. Protocols for DNA extraction and amplification follow Ballarin and Eguchi (2023). The standard DNA barcode (= the Folmer region) of the mitochondrial gene Cytochrome c oxidase subunit I (COI) was amplified using the universal primers pair LCO1490 and HCO2198 (Folmer et al. 1994). To ensure clear differences between the species groups and in particular the newly established *yambaruensis* group, we also amplified the nuclear gene Histone 3 (H3) using the primer pair H3aF and H3aR (Colgan et al. 1998). Additional *Solenysa* sequences for the genes COI and H3 were harvested from GenBank (https://www.ncbi.nlm.nih.gov/genbank/). The complete list of sequences used in this work is reported in Table 1.

**Table 1.** List of the *Solenysa* species and related GenBank accession codes used in the phylogenetic analysis. Asterisks refer to newly amplified sequences.

Code	Species	COI	H3	Locality	Notes
	*Agynetaramosa*	MZ610702	FJ838740		outgroup
Lin08	*S.bilamellata* sp. nov.	PQ872900*	na	Ōita Pref., Kyushu, Japan	
Lin38	*S.bilamellata* sp. nov.	PQ872906*	na	Ōita Pref., Kyushu, Japan	
	*S.lanyuensis*	OL693167	OL702838	Taiwan	
	*S.longqiensis*	KT002782	KT002883	Fujian Prov. PR China	
	*S.macrodonta*	OL693169	OL702840	Shimane Pref., Western Honshu, Japan	
	S.cf.macrodonta	KT002786	KT002887	Shimane Pref., Western Honshu, Japan	Reported as *S.reflexilis* A in GenBank, and as *S.reflexilis* in Tian et al. 2022, probably misidentification
Lin39	*S.macrodonta*	PQ872907*	na	Hiroshima Pref., Western Honshu, Japan	
	*S.mellotteei*	KT002781	KT002882	Kanagawa Pref., Central Honshu, Japan	
Lin64	*S.mellotteei*	PQ872910*	na	Tokyo Pref., Central Honshu, Japan	
	*S.ogatai*	OL693168	OL702839	Aichi Pref., Central Honshu, Japan	
	*S.partibilis*	KT002784	KT002885	Shiga Pref., Central Honshu, Japan	
	*S.protrudens*	KT002785	KT002886	Zhajiang Prov., PR China	
	S.cf.protrudens	GU338667	na	PR China	Reported as *Solenysa* sp. 14 IZCL110 in GenBank
	*S.reflexilis*	KT002787	KT002888	Kumamoto Pref., Kyushu, Japan	
Lin46	*S.reflexilis*	PQ872908*	na	Yakushima Is., Ryukyus, Japan	
	*S.retractilis*	KT002788	KT002889	Sichuan Prov., PR China	
	S.cf.retractilis	GU338658	na	PR China	Reported as *Solenysa* sp. 14 IZCL56 in GenBank
	*S.tianmushana*	KT002789	KT002890	Zhejinag Prov., PR China	
Lin18	*S.shimatchu* sp. nov.	PQ872901*	PQ879705*	Minami-Ōshima Is., Ryukyus, Japan	
Lin19	*S.shimatchu* sp. nov.	PQ872902*	PQ879706*	Minami-Ōshima Is., Ryukyus, Japan	
Lin20	*S.shimatchu* sp. nov.	PQ872903*	PQ879707*	Minami-Ōshima Is., Ryukyus, Japan	
Lin63	*S.trunciformis*	PQ872909*	na	Tokushima Pref., Shikoku, Japan	
	S.cf.trunciformis	KT002783	KT002884	Shikoku, Japan	Reported as *S.mellotteei* B in GenBank, probably misidentification
	*S.wulingensis*	KT002790	na	Hunan Prov., PR China	
Lin21	*S.yambaruensis* sp. nov.	PQ872904*	PQ879708*	Okinawa Is., Ryukyus, Japan	
Lin22	*S.yambaruensis* sp. nov.	PQ872905*	PQ879709*	Okinawa Is., Ryukyus, Japan	
	*S.yangmingshana*	OL693166	OL702837	Taiwan	

Sequences were visually checked and aligned using the online version of MAFFT software v. 7 (https://mafft.cbrc.jp/alignment/server/) under the G-INS-I method and subsequently translated to proteins using MEGA X v. 10.0.5 (Kumar et al. 2018) to check for potential stopping codons. The species *Agynetaramosa* Jackson, 1912, a basal Erigoninae Emerton, 1882, was selected as outgroup due to the proximity of this genus with *Solenysa* [see for example Ballarin and Yamasaki (2021: fig. 5) or Tian et al. (2022)]. When both gene fragments were available for the same individual, COI and H3 sequences were concatenated in a single sequence.

We conducted two distinct phylogenetic analyses, one using a maximum likelihood (ML) analysis in RAxML-NG (Kozlov et al. 2019) and another with a Bayesian Inference (BI) analysis in MrBayes v. 3.2.7 (Ronquist et al. 2012). Both analyses were run remotely on CIPRES Science Gateway v. 3.3 (https://www.phylo.org/). Partition and model were tested under the corrected Akaike’s Information Criterion (AICc). using the model selection option in the online version of IQtree Web Server (http://iqtree.cibiv.univie.ac.at/). Following the results the sequence dataset was partitioned by gene with the COI 3^rd^ codon considered separately.

ML analysis was performed under a rapid bootstrap of 1,000 replicates with a GTRGAMMAI model and the standard parameters suggested by the RAxML software. BI was performed running four Monte Carlo Markov chains (MCMCs) for one million generations with a 25% burning fraction using the substitution models suggested by IQtree: invgamma for both gene partitions and gamma for the COI 3^rd^ codon. Trees were sampled every 1,000 generations. TRACER v. 1.7.1 (Rambaut et al. 2018) was used to check that the effective sample size and consequent chains convergence were properly reached (ESS > 200). The Figtree software v. 1.4.3 (http://tree.bio.ed.ac.uk/software/ﬁgtree/) was used to graphically represent the phylogenetic trees. Nodes with ML bootstrap value (BV) ≥ 75 or BI posterior probability (PP) ≥ 0.95 were considered highly supported, while BV ≥ 70 or PP ≥ 0.90 were considered middle supported.

An uncorrected pairwise-distance genetic divergence analysis was carried out in MEGA X to test the genetic variability among the species and within each species group. We created a reduced dataset by selecting a single sequence of the COI barcode for each species. Interspecific divergence was calculated under a bootstrap method with 1,000 replications and all the other options set as default.

## ﻿Results

### ﻿Taxonomic account

**Family Linyphiidae Blackwall, 1859**

#### ﻿Genus Solenysa

Taxon classificationAnimaliaAraneaeLinyphiidae

Simon, 1894

E4C3CE52-F95D-5844-9ED7-F0B961A9C60E

##### Type species.

*Solenysamellotteei* Simon, 1894; type locality: Japan.

##### Distribution.

East Asia: Eastern mainland China, Taiwan, Korea, Japan (mainland and Ryukyus, absent in Hokkaido).

***Solenysayambaruensis* group sensu Ballarin & Eguchi**

**Composition.** Two species, *S.yambaruensis* sp. nov.; *S.shimatchu* sp. nov.

**Diagnosis.** Males of the *S.yambaruensis* group can be separated from males of other species groups by the following combination of characters: a well-developed, protruding proximal tibial apophysis (PTP) bearing three robust spines (vs PTP reduced or bearing thinner setae); a cymbium with a massive probasal cymbial apophysis (PBP) strongly bent and concave to form a wide pocket, lacking any clear spurs (vs PBP less developed, or with a different shape, or bearing some spurs); a paracymbium (P) elongated dorsal-ventrally with a well-developed ventral lobe (vs P differently shaped, elongated antero-posteriorly or with a reduced ventral lobe); a protruding, lobated median terminal apophysis (MTA) longer than wide and lacking any clear protrusions (vs MTA wider than long, or with a different shape and having some protrusions); a lamella with three, uniquely shaped branches (LA1−3) all well-developed: LA1 ribbon-like and transparent, LA2 strongly sclerotized, needle-like, LA3 with a wide, ribbon-like basal part and ending with a sclerotized, needle-like tip (vs LA1−3 less sclerotized, or with a different shape).

Females of the *S.yambaruensis* group can be distinguished from females of other species groups by the following combination of characters: solenoid (SL) with a smooth surface folded into two transversal coils (vs SL differently shaped, with a wrinkled surface, lacking two clear wide folds); a dorsal plate (DP) with a short, undivided rectangular lobe (LDP) protruding posteriorly (vs LDP lacking, divided into two lobes, or differently shaped).

**Description.** Cephalic area distinctly elevated in both sexes. Carapace oval with conspicuous lateral lobes. Carapace, chelicera, mouth parts, and sternum uniformly brick-red. Chelicera with four promarginal and three retromarginal teeth. Legs uniformly red-yellowish. TmI = 0.54, TmIV absent. Leg Tibial spine formula = 1-1-1-1. Opisthosoma uniformly greyish with one or three white marks on dorsal side, one mark always on dorsal-posterior tip of opisthosoma. Other somatic features as in other *Solenysa* spp.

Palpal tibia elongated, ~ 2× longer than patella, bearing three long, thin setae on anterior-retrolateral side; proximal tibial apophysis (PTP) well-developed, bearing three robust spines. Cymbium with well-developed probasal cymbial apophysis (PBP), massive, folded to form a wide pocket strongly bent retrolaterally, hook-like when observed dorsally. Cymbial retrolateral process (CRP) thorn-like. Paracymbium (P) U-shaped, elongated dorsal-ventrally, ventral lobe (VLP) wide. Solenysa tegular triangle (STT) long and narrow. Lamella with three well-developed branches: anterior branch (LA1) ribbon-like, transparent; median branch (LA2) straight, strongly sclerotized, needle-like; posterior branch (LA3) with a more or less wide, ribbon-like basal part, ending with a sharp, sclerotized point, straight or bent anteriorly. Radix (R) strongly sclerotized. Distal suprategular apophysis (DSA) well-developed, strongly sclerotized. Median terminal apophysis (MTA) lobated, ~ 2× longer than wide, protruding antero-ventrally. Anterior terminal apophysis (ATA) ribbon-like, flattened, slightly twisted, and bent ventrally, ending with a more or less sharp tip, bearing a short median tooth (MT). Embolus (E) transparent, ribbon-like, and fringed.

Epigyne more or less protruding when observed laterally, Solenoid (S) with smooth surface lacking wrinkles, connected to the dorsal base of epigyne, folded anterodorsally with approximately two folds. Ventral plate (VP) V-shaped or trapezoidal; anterior border strongly concave, posterior border flat or slightly V-shaped. Dorsal plate (DP) undivided, bearing a more or less protruding lobe (LDP). Copulatory ducts (CD) thick, heading anteriorly then posteriorly before reaching spermathecae. Fertilization ducts (FD) thin, slightly twisted, bent anteriorly. Spermathecae (S) wide, oval.

**Distribution.** Ryukyu Archipelago

**Remarks.** The *Solenysa* species were grouped by Tu and Hormiga (2011) into four species groups based on the morphology of their genitalia: the *Solenysalongqiensis* group (*S.longqiensis*, *S.yangmingshana*, and *S.spiralis* (?) from Mainland China and Taiwan), the *S.wulingensis* group (*S.geumoensis*, *S.retractilis*, *S.wulingensis*, and *S.tianmushana* from Mainland China and Korea), the *S.protrudens* group (*S.protrudens* and *S.lanyuensis* from Mainland China and Taiwan), and the *S.mellotteei* group (*S.macrodonta*, *S.mellotteei*, *S.ogatai*, *S.partibilis*, *S.reflexilis*, and *S.trunciformis*, all from Japan) (Tu and Hormiga 2011; Tian and Tu 2018). Later molecular studies supported the monophyly of these groups. (Tian et al. 2022).

The two new species from the Central Ryukyus exhibit a unique combination of morphological characters that set them apart from the previously known species groups. Our molecular analysis further supports this distinction placing these species in a monophyletic clade, separated from the other existing species groups (Fig. 12). Based on these findings, we propose a new species group, the *Solenysayambaruensis* group, to accommodate the two Ryukyuan species.

#### ﻿ Solenysa shimatchu

Taxon classificationAnimaliaAraneaeLinyphiidae

Ballarin & Eguchi sp. nov.

FA6E43E5-7A8F-552B-B800-96FEFDADBB39

https://zoobank.org/041CD89C-4412-40D9-A56B-A9382A71FDAD

**Figure 1.**
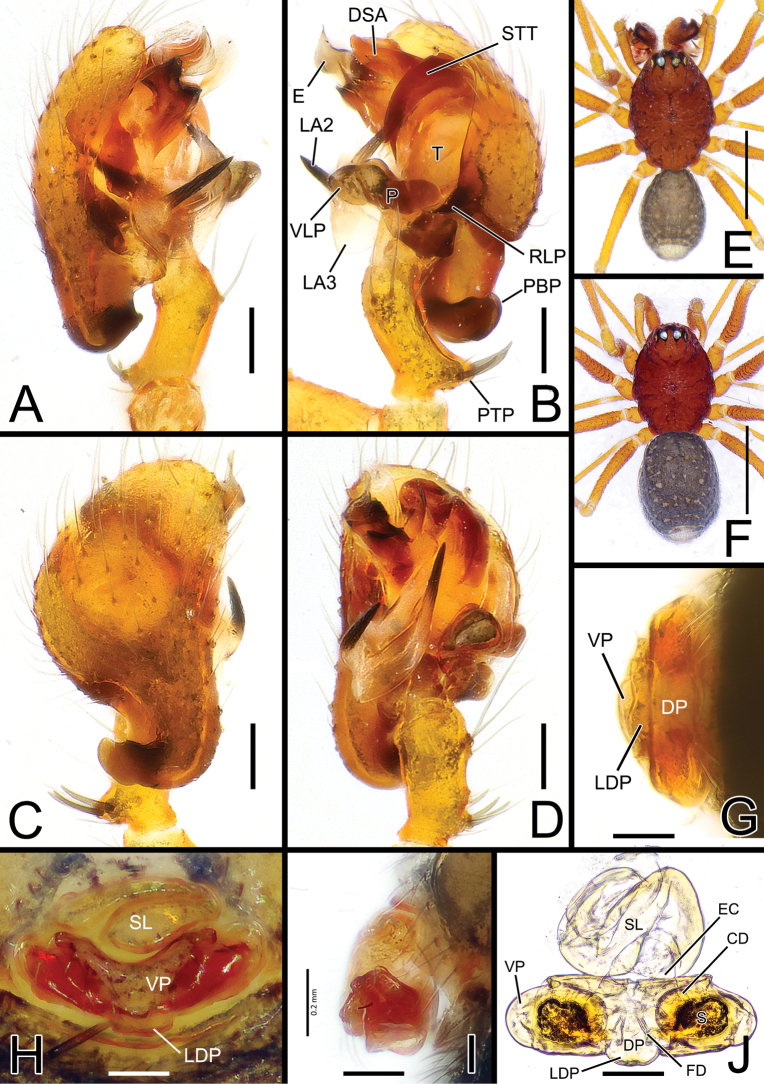
*Solenysashimatchu* sp. nov. **A** male palp, prolateral **B** ditto, retrolateral **C** ditto, dorsal **D** ditto, ventral **E** habitus of male, dorsal **F** habitus of female, dorsal **G** epigyne, posterior **H** ditto, ventral **I** ditto, lateral **J** vulva, dorsal. Abbreviations: DP dorsal plate; DSA distal suprategular apophysis; E embolus; EC epigynal collar; LA2 median branch of lamella; LA3 posterior branch of lamella; LDP lobe of the dorsal plate; P paracymbium; PBP probasal cymbial apophysis; PTP proximal tibial apophysis; RLP cymbial retrolateral process; S spermatheca; SL solenoid; STT*Solenysa* tegular triangle; T tegulum; VLP ventral lobe of P; VP ventral plate. Scale bars: 0.05 mm (**A–D, G–J**); 0.5 mm (**E, F**).

Figs 1A–J , 4A−C , 5A, B

##### Material examined.

***Holotype* ♂ Japan**: • **Kagoshima Pref.**, Amami-Ōshima Is., Setouchi, Amurogama, 121 m, 28.22261°N, 129.31695°E, humid forest litter in a flat area near a creek, 10.Jul.2021, F. Ballarin leg. (NSMT-Ar26184). ***Paratypes*. Japan: Kagoshima Pref.**, Amami-Ōshima Is., • 3 ♀, Amami, Naze Oaza Asato, 176 m, 28.33066°N, 129.48115°E, forest litter, 8.Jul.2021, F. Ballarin leg. (NSMT-Ar26185) • 1 ♀, Sumiyocho Oaza Kawauchi, 54 m, 28.31219°N, 129.42390°E, forest litter on a steep cliff, 8.Jul.2021, F. Ballarin leg. (NSMT-Ar26186) • 1 ♂, Yamato, Ongachi, ~ 28.33111°N, 129.39436°E, 9.Mar.2014, T. Suguro leg. (TKPM-AR3243) • 1 ♂, 3 ♀, Yamato, Tsunagu, 196 m, 28.33224°N, 129.41763°E, humid forest litter with stones near a creek, 9.Jul.2021, F. Ballarin leg. (MSNVR-Ar032–035) • 2 ♀, Naze, Oaza Chinase, 63 m, 28.34896°N, 129.44969°E, broadleaf forest litter, 9.Jul.2021, F. Ballarin leg. (TKPM-AR3244) • 1 ♂, 5 ♀, Setouchi, Amurogama, 121 m, 28.22261°N, 129.31695°E, humid forest litter in a flat area near a creek, 10.Jul.2021, F. Ballarin leg. (MNHAH) • 1 ♂, 1 ♀, Tatsugo, Akina, 245 m, 28.42290°N, 129.54688°E, rather dry forest litter, F. Ballarin leg. (FBPC) • 2 ♀, Uken, Ashiken, 208 m, 28.30780°N, 129.27311°E, rather dry litter on a steep slope near a creek, 12.Jul.2021, F. Ballarin leg. (NSMT-Ar26187).

##### Other material examined.

**Japan: Kagoshima Pref.**, Amami-Ōshima Is., • 1 ♀, Naze, Oaza Chinase, same locality and date, 274 m, 28.35705°N, 129.45436°E, broadleaf forest litter, 9.Jul.2021, F. Ballarin leg. (FBPC) • 1 ♀, Tatsugo, Akina, 64 m, 28.42192°N, 129.55104°E, humid forest litter with stones, 11.Jul.2021, F. Ballarin leg. (FBPC) • Tokunoshima Is., 1 ♂, 1 ♀, Amagi-cho, Nishiagina, ~ 27.7639°N, 128.9398°E, 28.Mar.2018, T. Suguro leg. (NSMT)

##### Diagnosis.

**Figure 2.**
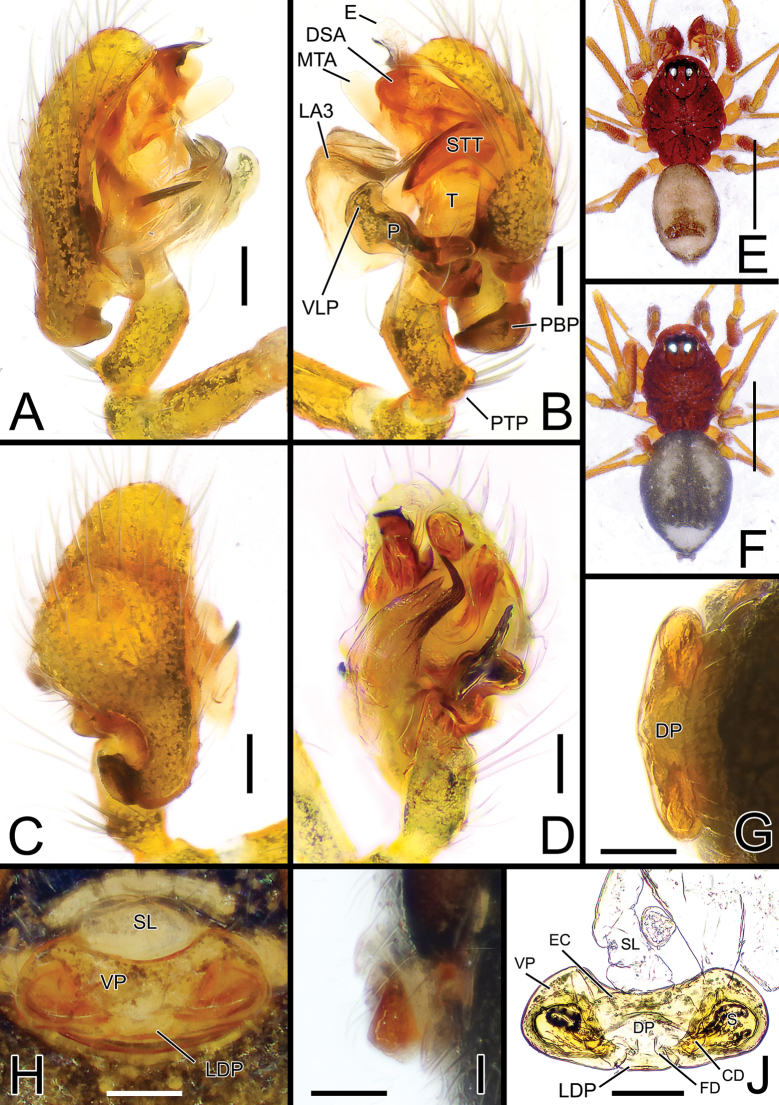
*Solenysayambaruensis* sp. nov. **A** male palp, prolateral **B** ditto, retrolateral **C** ditto, dorsal **D** ditto, ventral **E** habitus of male, dorsal **F** habitus of female, dorsal **G** epigyne, posterior **H** ditto, ventral **I** ditto, lateral **J** vulva, dorsal. Abbreviations: CD copulatory duct; DP dorsal plate; DSA distal suprategular apophysis; E embolus; EC epigynal collar; FD fertilization duct; LA3 posterior branch of lamella; LDP lobe of the dorsal plate; MTA median terminal apophysis; P paracymbium; PBP probasal cymbial apophysis; PTP proximal tibial apophysis; S spermatheca; SL solenoid; STT*Solenysа* tegular triangle; T tegulum; VLP ventral lobe of P; VP ventral plate. Scale bars: 0.05 mm (**A–D, G–J**); 0.5 mm (**E, F**).

Species closely related to *S.yambaruensis* sp. nov. from which it can be easily separated by the dorsal color pattern of the opisthosoma, having a single white mark on the dorsal-posterior tip of the opisthosoma (vs three marks) (cf. Fig. 1A−E, F vs Fig. 2E, F). Males of *S.shimatchu* sp. nov. can be easily separated from males of *S.yambaruensis* sp. nov. by the different shape of the posterior branch of the lamella (LA3), thinner and ending with a long and straight single needle-like tip (vs LA3 wider, ribbon-like, and ending with a bent spine and two denticles) (cf. Fig. 4A−C vs Fig. 4D−F). Additionally, the paracymbium (P) has a less-developed and shorter ventral lobe (VLP), headed ventrally (vs VLP with a wider lobe headed antero-ventrally); the probasal cymbial apophysis (PBP) is less bent and headed retrolaterally when observed dorsally (vs PBP more bent and headed antero-retrolaterally); and the proximal tibial apophysis (PTP) is more developed and with thicker spines (vs PTP less developed and with thinner spines) (cf. Fig. 1A−D vs Figs 2A−D, 5A, B, C–F).

Females of *S.shimatchu* sp. nov. can be distinguished from females of *S.yambaruensis* sp. nov. by the different shape of the epigyne: more rounded and protruding when observed laterally (vs flatter and less protruding); having a more V-shaped ventral plate (VP) (vs VP more trapezoidal with flatter posterior borders); and a more protruding lobe of the dorsal plate (LDP) (vs LDP less protruding) (cf. Fig. 1G−J vs Fig. 2G−J).

For additional separation from other *Solenysa* spp. see the species group’s diagnosis.

##### Description.

Male (holotype). Total length: 1.21. Prosoma 0.71 long; 0.46 wide. Clypeus 0.24 long. Habitus as in Fig. 1E. Length of legs as follows: I 2.38 (0.62, 0.15, 0.63, 0.53, 0.45), II 2.02 (0.56, 0.14, 0.51, 0.43, 0.38), III 1.61 (0.43, 0.13, 0.37, 0.36, 0.33), IV 1.80 (0.50, 0.13, 0.44, 0.39, 0.34). Opisthosoma uniformly greyish with scattered small lighter dots, one wider, distinct dorsal whitish mark near posterior tip of opisthosoma. Other somatic characters as reported in species group.

Palp as in Figs 1A−D, 4A−C, 5A, B. Proximal tibial apophysis (PTP) strongly protruding antero-retrolaterally, bearing 3 thick spines. Probasal cymbial apophysis (PBP), massive, hook-like when observed dorsally, headed retrolaterally. Ventral lobe of paracymbium (VLP) headed ventrally. Lamella with 3 well-developed branches: LA1 wide, ribbon-like, transparent; LA2 and LA3 both sclerotized, strait, needle-like; LA2 shorter, headed antero-ventrally; LA3 longer, with a slightly wider, ribbon-like basal part, ending sharp and thin, headed antero-dorsally. Posterior terminal apophysis (PTA), thin and transparent, almost invisible. Median terminal apophysis (MTA) half-transparent, lobated, slightly twisted. Anterior terminal apophysis (ATA) ribbon-like, strongly sclerotized, flattened, slightly twisted, headed anteriorly, ending with a sharp tip. Median tooth (MT) of ATA small and stocky, headed anteriorly. Embolus (E) transparent, twisted, and fringed, partially hidden by ATA.

Female (one of the paratypes). Total length: 1.26. Prosoma 0.72 long; 0.46 wide. Clypeus 0.24 long. Habitus as in Fig. 1F. Length of legs as follows: I 2.22 (0.58, 0.16, 0.57, 0.50, 0.41), II 1.93 (0.53, 0.13, 0.47, 0.43, 0.37), III 1.61 (0.43, 0.14, 0.35, 0.36, 0.33), IV 1.95 (0.53, 0.14, 0.48, 0.43, 0.37). Color and other features as in male.

Epigyne and vulva as in Fig. 1G−J, strongly protruding when observed laterally. Solenoid (SL) folded anterodorsally with 2 wide transversal folds before reaching the dorsal base of epigyne. Ventral plate (VP) slightly V-shaped, anterior border strongly concave. Dorsal plate (DP) undivided, bearing a well-developed rectangular ventral lobe (LDP) protruding posteriorly. Copulatory ducts (CD) thick, heading antero-laterally then posteriorly before reaching posterior side of spermathecae. Fertilization ducts (FD) thin, Z-shaped, bent anteriorly. Spermathecae (S) wide, oval (Fig. 1J).

##### Etymology.

The specific name is derived from the word “shimatchu” (島っちゅ) meaning “islander” in the Amami-Ōshima local language. The name refers to the insular origin of the species endemic to Amami-Ōshima and Tokunoshima islands. Name in apposition.

##### Distribution.

Endemic to Amami-Ōshima and Tokunoshima islands, Central Ryukyus, Japan (Fig. 13).

##### Habitat.

This species has been observed building simple sheet webs in open spaces in humid leaf litter on the floor of broadleaved forest.

#### ﻿ Solenysa yambaruensis

Taxon classificationAnimaliaAraneaeLinyphiidae

Ballarin & Eguchi sp. nov.

9BAB341D-0369-5B00-8C5F-4130077CA5C0

https://zoobank.org/A990BEDD-90C6-47E4-B5AD-485D1899684B

Figs 2A–J , 4D−F , 5C–F

##### Material examined.

***Holotype* ♂. Japan**: • **Okinawa Pref.**, Okinawa Is., Kunigami Distr., Kunigami Vill., Yona, Yambaru National Park, 185 m, 26.74755°N, 128.22347°E, humid forest litter, 25.Feb.2021, F. Ballarin leg. (NSMT-Ar26188). ***Paratypes*. Japan: Okinawa Pref.**, Okinawa Is., • 1 ♀, Kunigami Distr., Kunigami Vill., Yona, Yambaru National Park, 185 m, 26.74755°N, 128.22347°E, humid forest litter, 25.Feb.2021, F. Ballarin leg (NSMT-Ar26189) • 3 ♀, same locality, 46 m, 26.7601°N, 128.2190°E, 03.Sep.2023, humid forest leaf litter along the road, tullgren funnel, R. Itou, R. Kaneko, Y. Hiruma, and K. Watanabe leg. (MNHAH) • 2 ♀, same locality, 240 m, 26.74335°N, 128.22608°E, humid broadleaf litter near a small creek, 02.Sep.2024, F. Ballarin leg. (FBPC) • 1 ♂, Sate, 80 m, 26.78245°N, 128.22061°E, broadleaf forest litter on a slope with rocks, 13.May.2022, F. Ballarin leg. (FBPC) • 1 ♂, 3 ♀, same locality, 03.Sep.2023, F. Ballarin leg. (NSMT-Ar26190) • 1 ♂, 1 ♀, Ginama, 190 m, 26.82804°N, 128.25471°E, broadleaf forest litter, 04.Sep.2024, F. Ballarin leg. (TKPM-AR3245) • 1 ♂, 1 ♀, same locality, 210 m, 26.82743°N, 128.25885°E, pine forest litter, 04.Sep.2024, F. Ballarin leg. (MSNVR-Ar036–037) • 3 ♀, Oku, Ryukyu University Okuyamaso, 26.8365°N, 128.2715°E, 03.Sep.2023, forest leaf litter, sifter, R. Itou, R. Kaneko, Y. Hiruma, and K. Watanabe leg. (MNHAH) • 1 ♂, Nago, 10.Nov.2018, A. Tanikawa leg. (MNHAH)

##### Other material examined.

**Japan: Okinawa Pref**., Okinawa Is., • 1 ♀, Nago, 13.Nov.2007, A. Tanikawa leg. (MNHAH); • 1 ♀, same locality, 6.Nov.2009, A. Tanikawa leg. (MNHAH) • 1 ♀, same locality, 8.Dec.2009, A. Tanikawa leg. (TKPM) • 1 ♀, same locality, 21.Oct.2010, A. Tanikawa leg. (TKPM) • 1 ♂, same locality, 15.Apr.2011, A. Tanikawa leg. (TKPM) • 1 ♂, 1 ♀, Onna, Tancha, near OIST campus, 140 m, 26.45947°N, 127.83674°E. mixed forest litter along the road, 30.Aug.2023, F. Ballarin leg. (FBPC) • 1 ♂, Kunigami Distr., Kunigami Vill., Hama, Near Nagao-Bashi Bridge, 26.70897°N, 128.19708°E, 2.Jan.2024, forest litter, Z. Touyama leg. (ZTPC) • 1 ♂, 1 ♀, same locality, 20.Mar.2024, Z. Touyama & R. Yamauchi leg. (ZTPC) • 1 ♀, Hama, 26.70130°N, 128.19685°E, 2.Jan.2024, Z. Touyama leg. (ZTPC) • Kumejima Is., 1 ♀, Shimajiri-gun, Maja, 95 m 26.34819°N, 126.80254°E, litter in a broadleaf forest, 18.May.2022, F. Ballarin leg. (FBPC).

##### Diagnosis.

See the diagnosis of *S.shimatchu* sp. nov. above.

##### Description.

Male (holotype). Total length: 1.24. Prosoma 0.68 long; 0.47 wide. Clypeus 0.24 long. Habitus as in Fig. 2E. Length of legs as follows: I 2.62 (0.69, 0.15, 0.70, 0.59, 0.49), II 2.31 (0.64, 0.15, 0.57, 0.50, 0.45), III 1.81 (0.51, 0.14, 0.41, 0.40, 0.36), IV 2.21 (0.64, 0.14, 0.53, 0.48, 0.42). Opisthosoma uniformly greyish with three distinct, white-greyish marks on dorsal side: two wide parallel, elongated marks in the central-anterior part of opisthosoma, one wide mark near posterior tip. Marks partially fused together in some individuals. Other somatic characters as in species group.

Palp as in Figs 2A−D, 4D−F, 5C–F. Proximal tibial apophysis (PTP) slightly protruding, bearing three spines. Probasal cymbial apophysis (PBP), massive, strongly bent, hook-like when observed dorsally, headed antero-retrolaterally. Ventral lobe of paracymbium (VLP) wide, laterally flattened, strongly protruding antero-ventrally. Lamella with three well-developed branches: LA1 transparent and wide, ribbon-like, half-twisted; LA2 and LA3 both sclerotized; LA2 thin, strait, needle-like, headed antero-ventrally; LA3 longer, basal part wide, ribbon-like, headed antero-retrolaterally, ending with two small denticles and one sharp needle-like tip bent with a 90° angle antero-dorsally. Posterior terminal apophysis (PTA), thin and transparent, headed anteriorly, clearly visible when the palp is observed ventrally. Median terminal apophysis (MTA) flattened, long and lobated, sightly sclerotized, strongly protruding antero-ventrally. Anterior terminal apophysis (ATA) ribbon-like, strongly sclerotized, flattened, slightly twisted, headed ventrally, ending with a blunt tip. Median tooth (MT) of ATA small, thorn-like with a sharp tip, headed posteriorly. Embolus (E) transparent, twisted, and fringed, partially hidden by ATA.

Female (one of the paratypes). Total length: 1.25. Prosoma 0.63 long; 0.44 wide. Clypeus 0.21 long. Habitus as in Fig. 2F. Length of legs as follows: I 2.42 (0.65, 0.15, 0.63, 0.52, 0.47), II 2.07 (0.52, 0.14, 0.51, 0.46, 0.44), III 1.73 (0.45, 0.12, 0.41, 0.37, 0.38), IV 2.06 (0.55, 0.14, 0.50, 0.45, 0.42). Color and other features as in male.

Epigyne and vulva as in Fig. 2G−J, flattened and only slightly protruding when observed laterally. Solenoid (SL) folded anterodorsally with 2 transversal folds before reaching the dorsal base of epigyne. Ventral plate (VP) trapezoidal with rounded lateral borders, anterior border concave, ventral border flattened. Dorsal plate (DP) undivided, bearing a short, rectangular ventral lobe (LDP) slightly protruding posteriorly. Copulatory ducts (CD) thick, heading anteriorly then posteriorly before reaching posterior side of spermathecae. Fertilization ducts (FD) thin, S-shaped, slightly twisted, bent anteriorly. Spermathecae (S) wide, oval (Fig. 2J).

##### Etymology.

The specific name is derived from the type locality area where this species was initially found, the Yambaru National Park. This renowned protected area covers the Northern portion of Okinawa Island and has been included in the UNESCO World Heritage List since 2021.

##### Distribution.

Endemic to Okinawa Honto and Kumejima islands, Central Ryukyus, Japan (Fig. 13).

##### Habitat.

Humid leaf litter on the floor of broadleaved and mixed forests.

***Solenysamellotteei* group sensu Tu & Hormiga (2011)**

**Composition.** Seven species: *Solenysamellotteei* Simon, 1894; *S.macrodonta* Wang, Ono & Tu, 2015; *S.ogatai* Ono, 2011; *S.partibilis* Tu, Ono & Li, 2007; *S.reflexilis* Tu, Ono & Li, 2007; *S.trunciformis* Wang, Ono & Tu, 2015; *S.bilamellata* sp. nov.

**Diagnosis.** See Tu and Hormiga (2011) and Wang et al. (2015).

**Description.** See Tu and Hormiga (2011) and Wang et al. (2015).

**Distribution.** Mainland Japan (Honshu, Shikoku, Kyushu, absent in Hokkaido).

#### ﻿ Solenysa bilamellata

Taxon classificationAnimaliaAraneaeLinyphiidae

Ballarin & Eguchi sp. nov.

9A892B8C-046E-5B3D-8EA1-463C2DBCE206

https://zoobank.org/4D81EE13-9BA8-4B82-AC37-75B93BAA8C8F

Figs 3A−J , 4G−I

##### Material examined.

***Holotype* ♂ Japan: Kyushu Is.**, • **Ōita Pref.**: Saiki City, Kitachi, Ono, 54 m, 32.92845°N, 131.94978°E, humid leaf litter in a mixed forest, 25.Mar.2019, F. Ballarin leg. (NSMT-Ar26191). ***Paratypes*. Japan**: • **Kyushu Is., Saga Pref.**: 1 ♀, Saga City, Kinryumachi Kinryu, Kotohira Shrine (金刀比羅神社), 33.330°N, 130.299°E, 31.Jul.2005, A. Akihisa leg. (NSMT-Ar26192) • **Ōita Pref.**: 1 ♂, 1 ♀, Saiki City, Kamae Oaza Kamaeura, 159 m, 32.81950°N, 131.91222°E, leaf litter in a deciduous broadleaf forest on a steep hill, 21.Mar.2019, F. Ballarin leg. (MNHAH) • 2 ♀, Kitachi, Ono, 54 m, 32.92845°N, 131.94978°E, humid leaf litter in a mixed forest, 25.Mar.2019, F. Ballarin leg. (NSMT-Ar26193) • 2 ♀, Kunisaki Peninsula, Kunisaki City, Akimachi Yagawa, 203 m, 33.49561°N, 131.60430°E, leaf litter in a broadleaf forest on a slope, 02.Aug.2022, sifter, F. Ballarin leg. (TKPM-AR3246).

##### Diagnosis.

**Figure 3.**
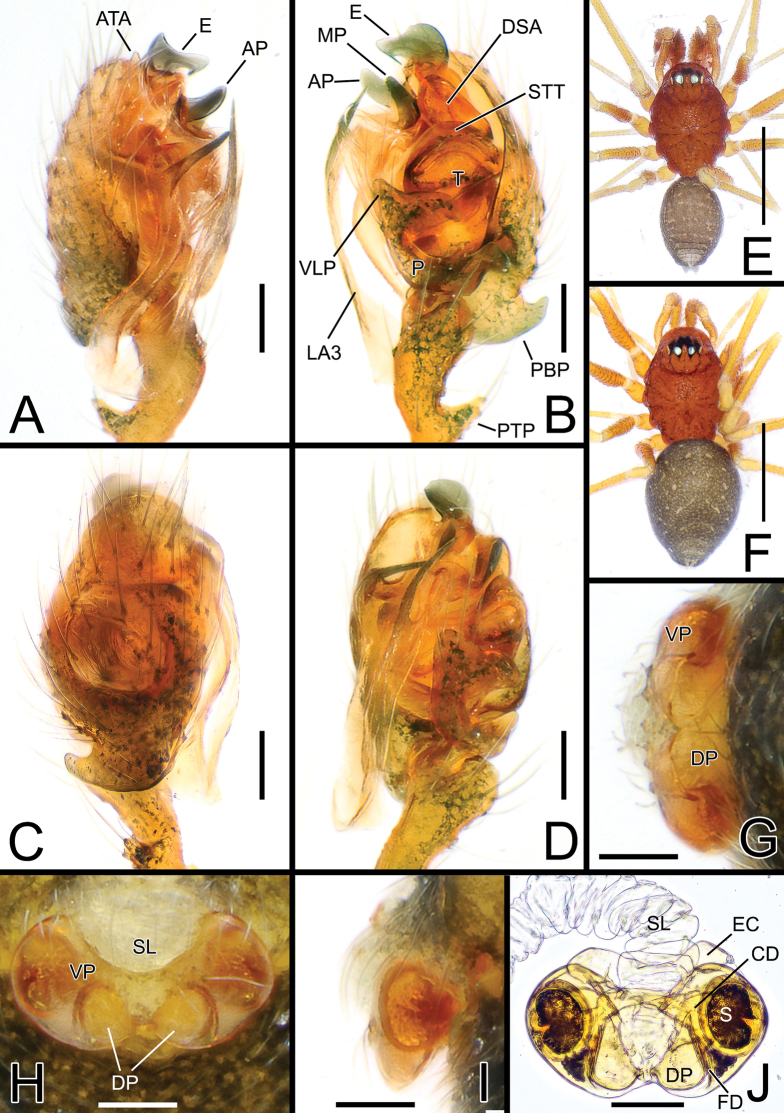
*Solenysabilamellata* sp. nov. **A** male palp, prolateral **B** ditto, retrolateral **C** ditto, dorsal **D** ditto, ventral **E** habitus of male, dorsal **F** habitus of female, dorsal **G** epigyne, posterior **H** ditto, ventral **I** ditto, lateral **J** vulva, dorsal. Abbreviations: AP anterior protrusion of MTA; CD copulatory duct; DP dorsal plate; DSA distal suprategular apophysis; E embolus; EC epigynal collar; FD fertilization duct; LA3 posterior branch of lamella; MTA median terminal apophysis; P paracymbium; PBP probasal cymbial apophysis; PP posterior protrusion of MTA; PTP proximal tibial apophysis; S spermatheca; SL solenoid; STT*Solenysa* tegular triangle; T tegulum; VLP ventral lobe of P; VP ventral plate. Scale bars: 0.05 mm (**A–D, G–J**); 0.5 mm (**E, F**).

**Figure 4.**
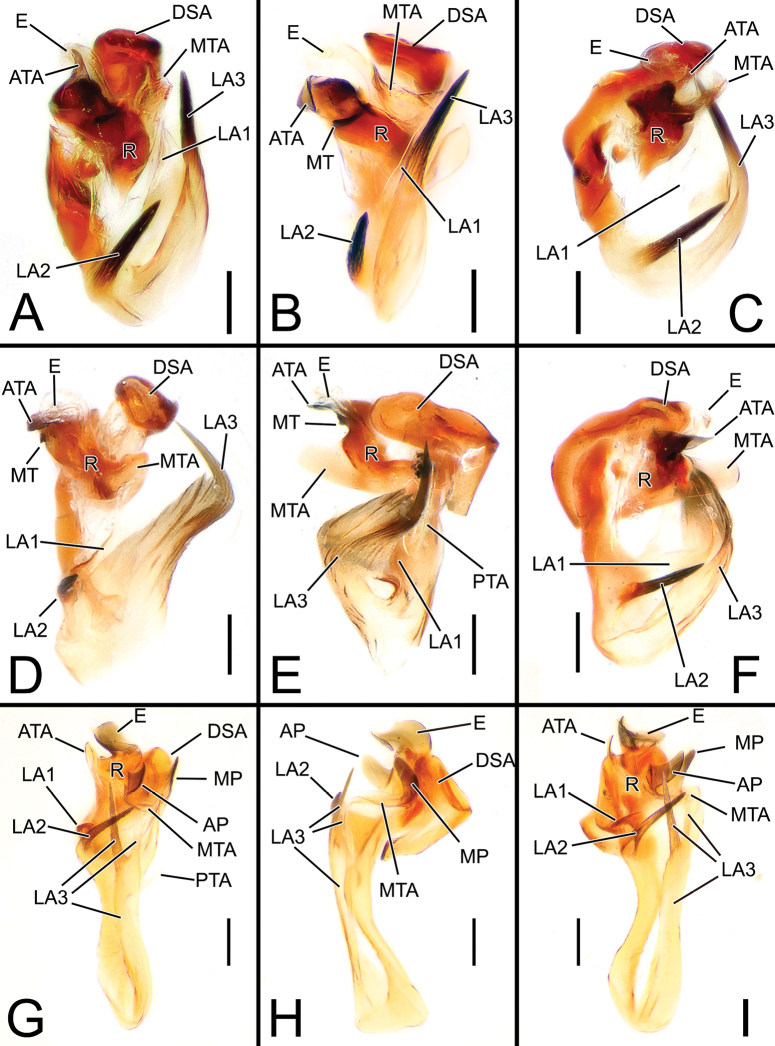
Embolic divisions of newly described *Solenysa* spp. **A** embolic division of *S.shimatchu* sp. nov., ventral **B** ditto, retrolateral **C** ditto, ventro-prolateral**D** embolic division of *S.yambaruensis* sp. nov., ventral **E** ditto, retrolateral **F** ditto, ventro-prolateral **G** embolic division of *Solenysabilamellata* sp. nov., ventral **H** ditto, retrolateral **I** ditto, ventro-prolateral. Abbreviations: AP anterior protrusion of MTA; ATA anterior terminal apophysis; DSA distal supra-tegular apophysis; E embolus; LA1 anterior branch of lamella; LA2 median branch of lamella; LA3 posterior branch of lamella; MT median tooth on anterior terminal apophysis; MP median protrusion of MTA; MTA median terminal apophysis; PTA posterior terminal apophysis; R radix. Scale bars: 0.05 mm.

Species closely related to other *Solenysa* species from western Japan. Males of *S.bilamellata* sp. nov. can be easily distinguished from males of *S.reflexilis*, *S.macrodonta*, and *S.trunciformis* by the presence of two large, lamellar-like protrusions of the median tegular apophysis (AP and MP) and having a rounded tip, both clearly visible when the palp is observed laterally (vs only AM visible and MP missing or reduced in *S.reflexilis*; or AM reduced and only MP clearly visible but with a pointed or truncated tip in the other three species). Additionally, *S.bilamellata* sp. nov. can be distinguished by the straight second branch of the lamella (LA2) (vs LA2 curved in *S.reflexilis* and *S.macrodonta*); by the smaller LA1 (LA1 longer in *S.reflexilis*); the sharper tip of the upper branch of LA3 (vs tip of LA3 wide and fringed in *S.macrodonta*), and by the shape of the lamellar-like anterior terminal hypophysis (ATA) (vs ATA with a different shape, wider in *S.reflexilis* or thinner in *S.macrodonta*, and *S.trunciformis*) (cf. Fig. 3A−D vs Figs 4G−I, 6A−D, 7A−D, 8A−D, 11A−F). Females of *S.bilamellata* sp. nov. can be distinguished from females of *S.reflexilis*, *S.macrodonta*, and *S.trunciformis* by the different shape of the epigyne, having a more transversely ovate ventral plate (VP) with short and pointed lateral borders headed internally and a dorsal plate (DP) with more rounded lobes (vs VP more trapezoidal with lateral borders headed externally and DP with more trapezoidal lobes in *S.reflexilis*; or VP more elongated posteriorly with longer lateral borders and DP with oval lobes in *S.macrodonta* and *S.trunciformis*) (cf. Fig. 3G−J vs Figs 6G−J, 7G−J, 8G−J).

**Figure 5.**
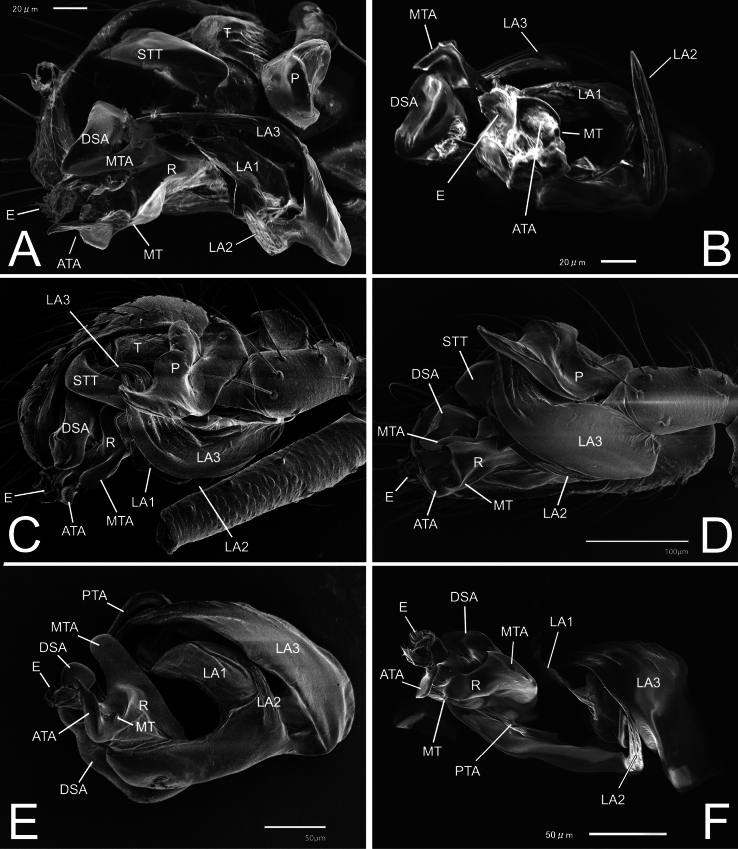
Palp and embolic division of *Solenysa* spp. under SEM microscope **A** palp of *S.shimatchu* sp. nov., ventro-retrolateral **B** ditto, embolic division, ventral **C** palp of *S.yambaruensis* sp. nov., ventro-retrolateral **D** ditto, ventral **E** ditto, embolic division, ventro-prolateral **F** ditto, ventral. Abbreviations: ATA anterior terminal apophysis; DSA distal supra-tegular apophysis; E embolus; LA1 anterior branch of lamella; LA2 median branch of lamella; LA3 posterior branch of lamella; MT median tooth on anterior terminal apophysis; MTA median terminal apophysis; P paracymbium; PTA posterior terminal apophysis; R radix; RLP cymbial retrolateral process; STTSolenysa tegular triangle; T tegulum.

##### Description.

Male (holotype). Total length: 1.22. Prosoma 0.67 long; 0.48 wide. Clypeus 0.24 long. Habitus as in Fig. 3E. Cephalic area distinctly elevated. Carapace oval with conspicuous lateral lobes. Carapace, chelicera, mouth parts, and sternum uniformly brick-red. Chelicera with four promarginal and three retromarginal teeth. Legs uniformly red-yellowish. Length of legs as follows: I (0.64, 0.15, 0.63, 0.52, 0.42), II (0.60, 0.14, 0.55, 0.44, 0.40), III (0.47, 0.14, 0.38, 0.36, 0.33), IV (0.55, 0.13, 0.51, 0.44, 0.37). TmI = 0.54, Opisthosoma uniformly greyish with scattered small white marks on dorsal side, dorsal-posterior tip of opisthosoma lighter gray in some individuals.

Palp as in Figs 3A−D, 4G−I. Palpal tibia elongated, ~ 2× longer than patella, bearing four long, thin setae on anterior-retrolateral side; proximal tibial apophysis (PTP) well-developed, strongly protruding dorsally, bearing four–five long, thin setae. Cymbium with well-developed probasal cymbial apophysis (PBP), hook-like when observed laterally or dorsally, bent retrolaterally, with massive basal part, ending with thin, rounded tip. Cymbial retrolateral process (CRP) thorn-like. Paracymbium (P) U-shaped, elongated antero-posteriorly, ventral lobe (VLP) protruding anteriorly. *Solenysa* tegular triangle (STT) thick. Lamella with three well-developed branches: anterior branch (LA1) short and stocky; median branch (LA2) straight, strongly sclerotized, needle-like; posterior branch (LA3) with long, ribbon-like basal part, distal part forked into two needle-like branches headed antero-dorsally, ventral branch sclerotized, dorsal branch transparent ending blunt. Radix (R) strongly sclerotized. Distal suprategular apophysis (DSA) well-developed, strongly sclerotized. Median terminal apophysis (MTA) bearing two well-visible, sclerotized, lamellar protrusions protruding antero-ventrally: anterior protrusion (AP) lobated, longer than wide; median protrusion (MP) also longer than wide and lobated but slightly thinner and sharper than AP, ending with a rounded, serrated tip. Anterior terminal apophysis (ATA) stocky, lobated, lacking any median tooths. Embolus (E) sclerotized, ribbon-like, twisted, ending with a sharp tip.

Female (one of the paratypes). Total length: 1.25. Prosoma 0.63 long; 0.45 wide. Clypeus 0.21 long. Habitus as in Fig. 3F. Length of legs as follows: I (0.61, 0.14, 0.60, 0.47, 0.40), II (0.56, 0.14, 0.50, 0.43, 0.38), III (0.44, 0.14, 0.35, 0.29), IV (0.58, 0.14, 0.48, 0.42, 0.37). Color and other features as in male.

Epigyne and vulva as in Fig. 3G−J, protruding, ventral side flattened when observed laterally. Solenoid (SL) with numerous small coils give a wrinkled texture, lacking clear, wide folds. Ventral plate (VP) transversely ovate, with pointed lateral borders headed posteriorly; anterior border strongly concave, posterior border rounded. Dorsal plate (DP) divided into two rounded lobes separated from each other by ~ 1/3 of their width. Copulatory ducts (CD) headed anteriorly then posteriorly before reaching spermathecae. Fertilization ducts (FD) thin, bent anteriorly. Spermathecae (S) wide, kidney-shaped (Fig. 3J).

##### Etymology.

The species name is derived from the Latin prefix *bi*- meaning “two,” and *lamellata* meaning “bearing lamellae.” It refers to the two flat, lamellar protrusions (AP and MP) of the median terminal apophysis that are diagnostic for this species. Adjective.

##### Distribution.

Endemic to northern and eastern Kyushu, Western Japan (Fig. 13). See also remarks of *S.reflexilis*.

##### Habitat.

Humid leaf litter on the floor of broadleaved forests.

#### ﻿ Solenysa reflexilis

Taxon classificationAnimaliaAraneaeLinyphiidae

Tu, Ono & Li, 2007

6E4C5A51-F3AE-52BC-9AA4-8F8254C2B7E6

Figs 6A−J , 11A, B

Solenysa reflexilis Tu et al., 2007: 58, fig. 1A–H (♂♀); Ono et al. 2009: 332, figs 1100–1104 (♂♀);
Wang et al. 2015: 52, fig. 4E, F (♀).

##### Material examined.

**Japan: Kyushu Is., Kagoshima Pref.**, • 1 ♀, Kirishima City, Kirishima Taguchi, Kinkowan National Park, 746 m, 31.86888°N, 130.89015°E, humid broadleaf forest litter, 29.Sep.2021, F. Ballarin leg. (FBPC) • 1 ♀, Kagoshima City, Yoshino Town, near Ryugamizu train station, 32 m, 31.64580°N, 130.60283°E, broadleaf forest litter on a slope, 22.Sep.2021, F. Ballarin leg. (FBPC) • Yakushima Is., 1 ♂, Onoaida, near Onoaida onsen, 121 m, 30.24178°N, 130.54786°E, rather dry broadleaf forest litter with stones, 24.Sep.2021, F. Ballarin leg. (FBPC) • 1 ♀, Koseda, 190 m, 30.38286°N, 130.62455°E, broadleaf forest litter on a gentle slope, 24.Sep.2021, F. Ballarin leg. (FBPC) • 1 ♀, same locality, 30.37402°N, 130.62608°E, mixed forest litter, 23.Sep.2021, K. Eguchi leg. (NSMT) • 1 ♀, Anbo, 207 m, 30.28454°N, 130.61799°E, broadleaf forest litter on a gentle slope, 24.Nov.2021, F. Ballarin leg. (NSMT) • 1 ♀, Kurio, 185 m, 30.29394°N, 130.42351°E, broadleaf forest litter on a gentle slope 25.Sep.2021, F. Ballarin leg. (FBPC).

##### Diagnosis.

See Tu et al. (2007).

##### Description.

**Figure 6.**
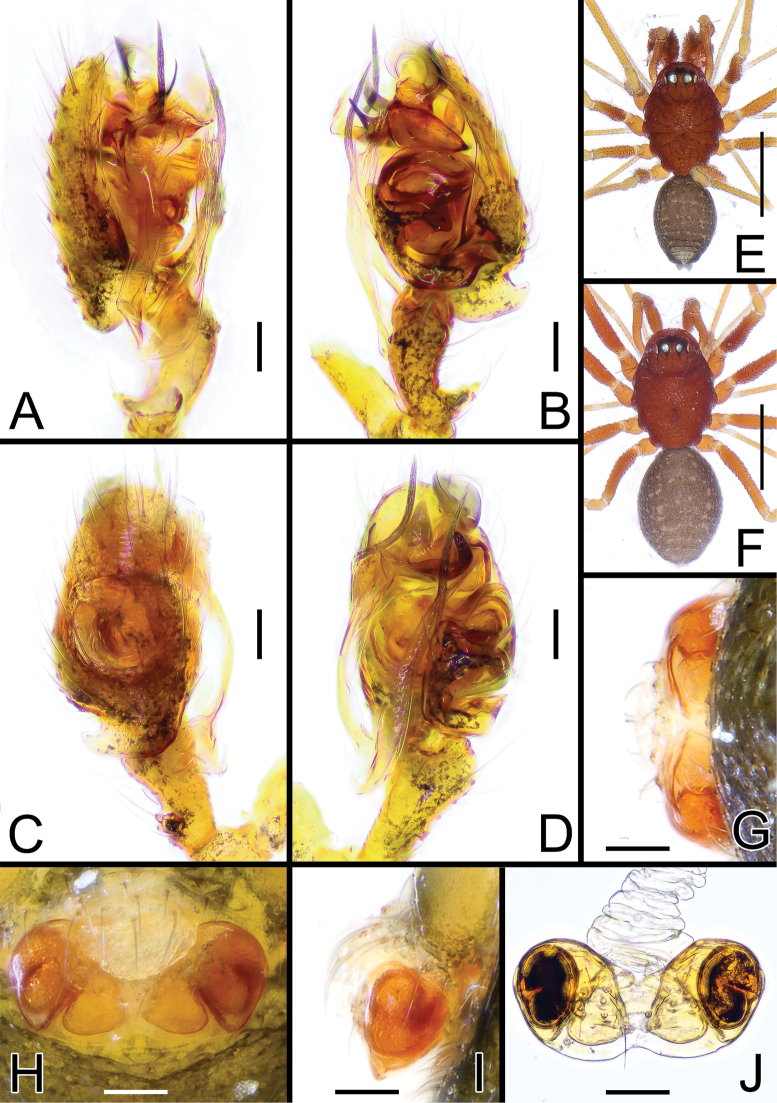
*Solenysareflexilis* from Yakushima **A** male palp, prolateral **B** ditto, retrolateral **C** ditto, dorsal **D** ditto, ventral **E** habitus of male, dorsal **F** habitus of female, dorsal **G** epigyne, posterior **H** ditto, ventral **I** ditto, lateral **J** vulva, dorsal. Scale bars: 0.05 mm (**A–D, G–J**); 0.5 mm (**E, F**).

Habitus of male as in Fig. 6E, habitus of female as in Fig. 6F. Palp as in Fig. 6A−D, embolic division as in Fig. 11A, B; epigyne and vulva as in Fig. 6G−J. See Tu et al. (2007) for a detailed description.

##### Type locality.

Shimo-kajiwara Itsuki-mura, Kuma-gun, Kumamoto Prefecture, Kyushu, Japan. (32.200°N, 130.500°E)

##### Remarks.

The current known distribution of *S.reflexilis* covers Kyushu (Nagasaki, Kumamoto, Ōita, and Kagoshima Prefectures) with additional records from Shikoku (Tokushima and Ehime Prefectures), and the Chugoku area in Western Honshu (Shimane Prefecture), Japan (Shinkai et al. 2024, this work) (Fig. 13). Our new data confirm the presence of *S.reflexilis* in southern Kyushu, particularly in mainland Kagoshima Prefecture and on the island of Yakushima, where this species has been recently reported by Shinkai and Tanikawa (2023). Previous records of this species in northern and eastern Kyushu should be replaced by the closely related *S.bilamellata* sp. nov. The record from Ōita prefecture by Serita (2022) might refer to a misidentification of the similar *S.bilamellata* sp. nov. Records from Shimane Prefecture (Hayashi et al. 2013a, b) and Shikoku Island (Tsurusaki et al. 2011, 2012; Bando 2015, 2019), all except one, predate the revision of the Japanese *Solenysa* species by Wang et al. (2015) and the description of *S.macrodonta* and *S.trunciformis*. Samples published by Bando (2015) examined by us all refer to *S.trunciformis*. Due to the possibility of misidentifications with other closely related and morphologically similar species endemic to the same areas, the presence of *S.reflexilis* in Eastern Kyushu, Western Honshu, and Shikoku, remains unclear and should be properly validated based on more recent evidence. Similarly, the precise boundaries between the ranges of *S.reflexilis* and *S.bilamellata* sp. nov. in Kyushu are unknown and should be addressed in more detail.

#### ﻿ Solenysa macrodonta

Taxon classificationAnimaliaAraneaeLinyphiidae

Wang, Ono & Tu, 2015

2FD77D5F-3F1B-5719-8BA2-51B41480D80F

Figs 7A−J , 11C, D

Solenysa macrodonta Wang et al., 2015: 48, figs 3A, 4C, D (♂♀).

##### Material examined.

**Japan: Hiroshima Pref.**, • 1 ♂, 6 ♀, Kure City, Yasuuracho Oaza Akozaka, 209 m, 34.31089°N, 132.72896°E, thick and rather dry broadleaf forest leaf litter on a steep slope, 04.Aug.2022, F. Ballarin leg. (FBPC).

##### Diagnosis.

See Wang et al. (2015).

##### Description.

**Figure 7.**
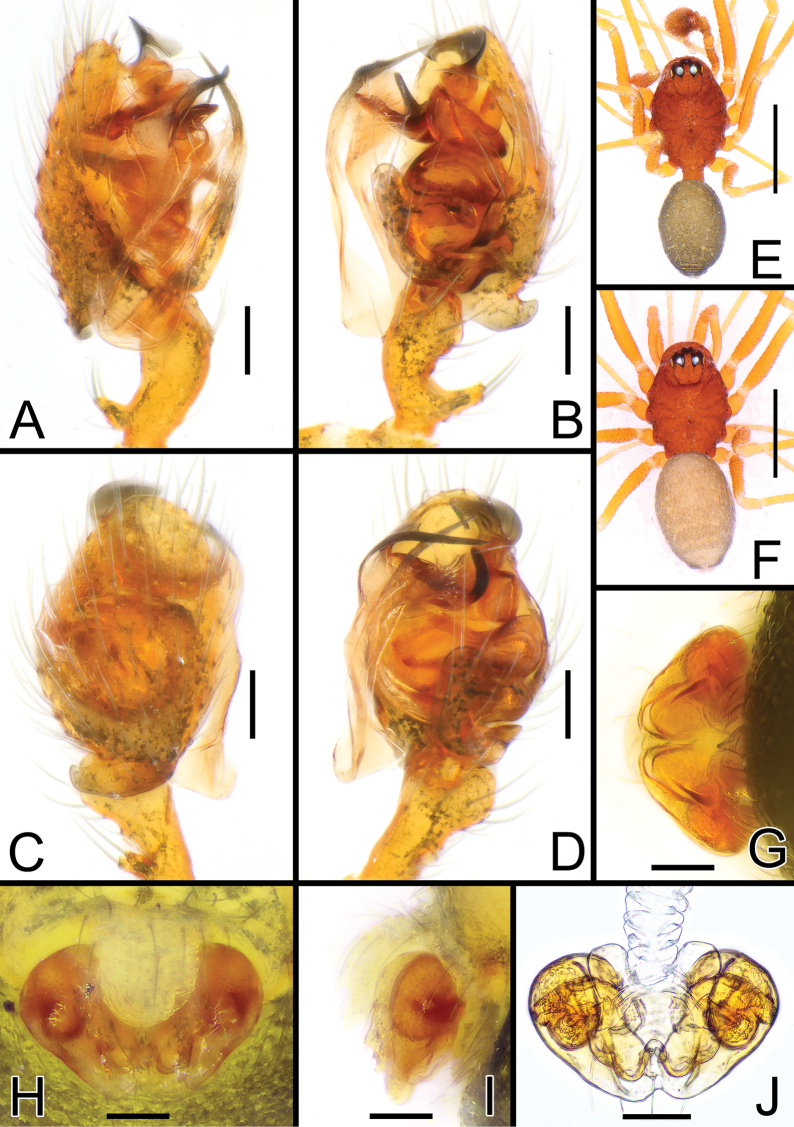
*Solenysamacrodonta* from Hiroshima **A** male palp, prolateral **B** ditto, retrolateral **C** ditto, dorsal **D** ditto, ventral **E** habitus of male, dorsal **F** habitus of female, dorsal **G** epigyne, posterior **H** ditto, ventral **I** ditto, lateral **J** vulva, dorsal. Scale bars: 0.05 mm (**A–D, G–J**); 0.5 mm (**E, F**).

Habitus of male as in Fig. 7E, habitus of female as in Fig. 7F. Palp as in Fig. 7A−D, embolic division as in Fig. 11C, D; epigyne and vulva as in Fig. 7G−J. See Wang et al. (2015) for a detailed description.

##### Type locality.

Nishida, Yunotsu City, Shimane Prefecture, Honshu, Japan (35.084°N, 132.401°E).

##### Remarks.

*Solenysamacrodonta* is distributed in the Chugoku area in Western Honshu with a few known records from Shimane and Okayama Prefectures (Shinkai et al. 2024). Here we report for the first time its presence in Hiroshima Prefecture (Fig. 13). Our male sample differs from the original description by having a shorter anterior protrusion (AP, central tooth in Wang et al. 2015) (cf. Fig. 11C, D vs Wang et al. 2015: fig. 6B). However, it shares the shape of the lamella with a transparent, upper branch of the LA2 ending with a wide, fringed tip. Additionally, we found no differences in the females’ morphology and the barcode of our samples matches those available in GenBank for this species collected in the Shimane Prefecture, the type area of the species (Fig. 12). Accordingly, we consider the differences in the male palp as possibly part of the intraspecific diversity of the species. Future comparisons with a larger number of individuals of *S.macrodonta* from different localities will help to clarify this issue.

#### ﻿ Solenysa trunciformis

Taxon classificationAnimaliaAraneaeLinyphiidae

Wang, Ono & Tu, 2015

065F8CA6-9719-578F-9EBD-2BAFEB12145A

Figs 8A−J , 11E, F

Solenysa mellotteei : Tu and Li 2006: 91, figs 21–30 (♂♀, misidentified per Wang et al. 2015: 54); Tu and Hormiga 2011: 499, figs 7B, 11H (♂♀, misidentified per Wang et al. 2015: 54).
S. trunciformis Wang et al., 2015: 54, figs 1A–D, 3B, 5E, F (♂♀).

##### Material examined.

**Japan: Shikoku Is., Tokushima Pref.**, • 3 ♂, 7 ♀, Myozai District, Kamiyama Town, Ano, Nashinoki-Toge pass, 11.Oct.1999, 33.91391°N, 134.28702°E, H. Bando leg. (TKPM) • 2 ♂, 3 ♀, Mima City, Tsurugi Town, Ichu, Tachinomoto, 33.94936°N, 134.06863°E, 1.Jan.2010, H. Bando leg. (TKPM) • 1 ♂, 5 ♀ (identified as *S.reflexilis* in Bando 2015), Anan City, Kamo Town, Omatsu-daigongen, 33.91466°N, 134.55208°E, 29.Aug.2013, H. Bando leg. (TKPM) • 1 ♀ (identified as *S.reflexilis* in Bando 2015), Anan City, Tsubachi Town, Toyono, 33.82427°N, 134.64894°E, 23.Sep.2013, H. Bando leg. (TKPM) • 1 ♂, 1 ♀, Anan City, Asebi Town, Arita, along road n°28, 33.87834°N, 134.55234°E, in the litter in a forest of sugi trees (*Cryptomeriajaponica*), 15.May.2019, F. Ballarin, T. and Yamasaki leg. (FBPC) • 1 ♂, 7 ♀, Tokushima City, Kamihachiman Town, Tatsumiyama, 34.01891°N, 134.51008°E, 18.Sep.2024, Y. Suzuki leg. (TKPM) • **Kochi Pref.**, 1 ♀, Muroto Peninsula, Motootsu, 243 m, 33.31339°N, 134.12262°E, deciduous forest leaf litter, 01.Mar.2022, F. Ballarin leg. (FBPC) • 1 ♂, Tosa City, Usachoryu, 33.42255°N, 133.45036°E, 15.Sep.2023, Y. Tsuji leg. (TKPM) • 1 ♂, Shimanto City, Gudo, 32.99045°N, 132.91411°E, 12.Jul.2023, Y. Tsuji leg. (TKPM) • **Okayama Pref.**, 1 ♂, Maniwa City, Shimoazae, near the entrance of Suwa-do cave (諏訪洞), 183 m, 34.97021°N, 133.62441°E, in the leaf litter of a deciduous forest 20.Apr.2019, F. Ballarin and T. Yamasaki leg. (FBPC) • 1 ♂, 1 ♀, Niimi City, Toyonagauyama, Safushi river’s valley (佐伏川), 254 m, 34.93934°N, 133.56580°E, in the litter of a deciduous forest on a very steep slope, 21.Apr.2019, F. Ballarin and T. Yamasaki leg. (MNHAH) • 1 ♀, Takahashi City, Kawakami Town, Kōyamaichi, Anatoyama Shrine (穴門山神社), 454 m, 34.74384°N, 133.39246°E, under stones near the shrine, 22.Apr.2019, F. Ballarin and T. Yamasaki leg. (FBPC).

##### Diagnosis.

See Wang et al. (2015).

##### Description.

**Figure 8.**
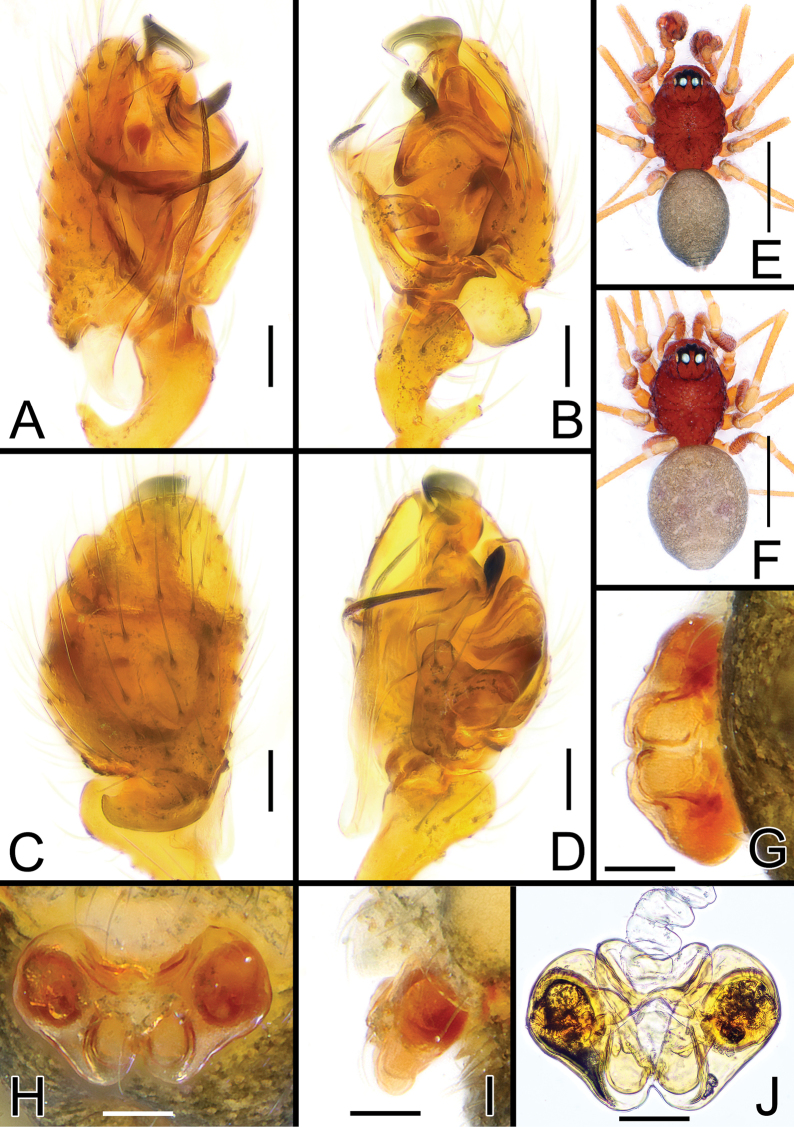
*Solenysatrunciformis***A** male palp, prolateral, sample from Okayama **B** ditto, retrolateral **C** ditto, dorsal **D** ditto, ventral **E** habitus of male, dorsal **F** habitus of female, dorsal **G** epigyne, posterior, sample from Tokushima **H** ditto, ventral **I** ditto, lateral **J** vulva, dorsal. Scale bars: 0.05 mm (**A–D, G–J**); 0.5 mm (**E, F**).

Habitus of male as in Fig. 8E, habitus of female as in Fig. 8F. Palp as in Fig. 8A−D, embolic division as in Fig. 11E, F; epigyne and vulva as in Fig. 8G−J. See Wang et al. (2015) for a detailed description.

##### Type locality.

Tondazaka, Shirahama City, Wakayama Prefecture, Honshu, Japan (33.625°N, 135.422°E).

##### Remarks.

The current distribution of this species covers Shikoku (Tokushima, Kanagawa, and Kochi Pref.), eastern Chugoku (Okayama Pref.), and southern Kansai (Wakayama Pref.), Japan (Shinkai et al. 2024, this work; Fig. 13). The male specimens from Okayama collected by us exhibits a massive and more squared median protrusion (MP) of the MTA together with a much shorter and triangular posterior protrusion (PP) when compared with the holotype (cf. Fig. 11E, F vs Wang et al. 2015: fig. 6F). The male from eastern Shikoku examined by us exhibits both the median (MP) and posterior (PP) protrusions strongly reduced. In all cases, the shape of the lamella remains constant. Due to the lack of specimens examined from the type locality area we cannot confirm if this is part of the intraspecific variability of the species or if *S.trunciformis* is a potential complex of multiple morphologically similar species. Yet, our preliminary molecular results suggest little genetic difference among the individual from Tokushima Pref. with the putative *S.trunciformis* from Shikoku harvested from GenBank (Fig. 12). Further investigations involving a molecular analysis of a larger number of individuals from different localities may help shed light on this matter.

###### ﻿*Solenysalongqiensis* group sensu Tu & Hormiga (2011)

#### ﻿ Solenysa longqiensis

Taxon classificationAnimaliaAraneaeLinyphiidae

Li & Song, 1992

6C0C42E9-3423-5B0E-839D-60514D753C20

Figs 9A−J , 11G, H

Solenysa longqiensis Li & Song, 1992: 6, fig. 1A–G (♂♀); Song et al. 1993: 861, fig. 17A–G (♂♀); Li et al. 1994: 80, figs 18, 19 (♀); Song et al. 1999: 204, fig. 116J, K, Q, R (♂♀); Tu and Li 2006: 91, figs 12–20 (♂♀); Tu and Hormiga 2011: 503, figs 7A, 14A–H, 15A–H (♂♀).

##### Material examined.

**Taiwan: Nantou County**, Ren’ai, • 1 ♀, Huisun Forest Area, 720 m, 24.09360°N, 121.03080°E, broadleaf forest litter, 09.July.2023, F. Ballarin leg. (TARI) • 1 ♂, same locality, 740 m, 24.08967°N, 121.03529°E, broadleaf forest litter along the trail, 12.July.2023, F. Ballarin leg. (TARI) • 1 ♂, 5 ♀, same locality, 685 m, 24.09295°N, 121.03247°E, broadleaf forest litter on a gentle slope, 13.July.2023, F. Ballarin leg. (NSMT) • 2 ♂, 4 ♀, same locality, 727 m, 24.09231°N, 121.03272°E, rather dry broadleaf forest litter, 14.July.2023, F. Ballarin leg. (FBPC) • 1 ♂, Menggu Waterfall, 910 m, 24.02824°N, 121.08067°E, broadleaf forest litter along the trail, 17.July.2023, F. Ballarin leg. (TARI).

##### Type locality.

Mt. Longqi, Yujiaping Town, Jiangle County, Fujian Province, China (26.700°N, 117.400°E)

##### Diagnosis.

See Tu and Li (2006) and Tu and Hormiga (2011).

##### Description.

**Figure 9.**
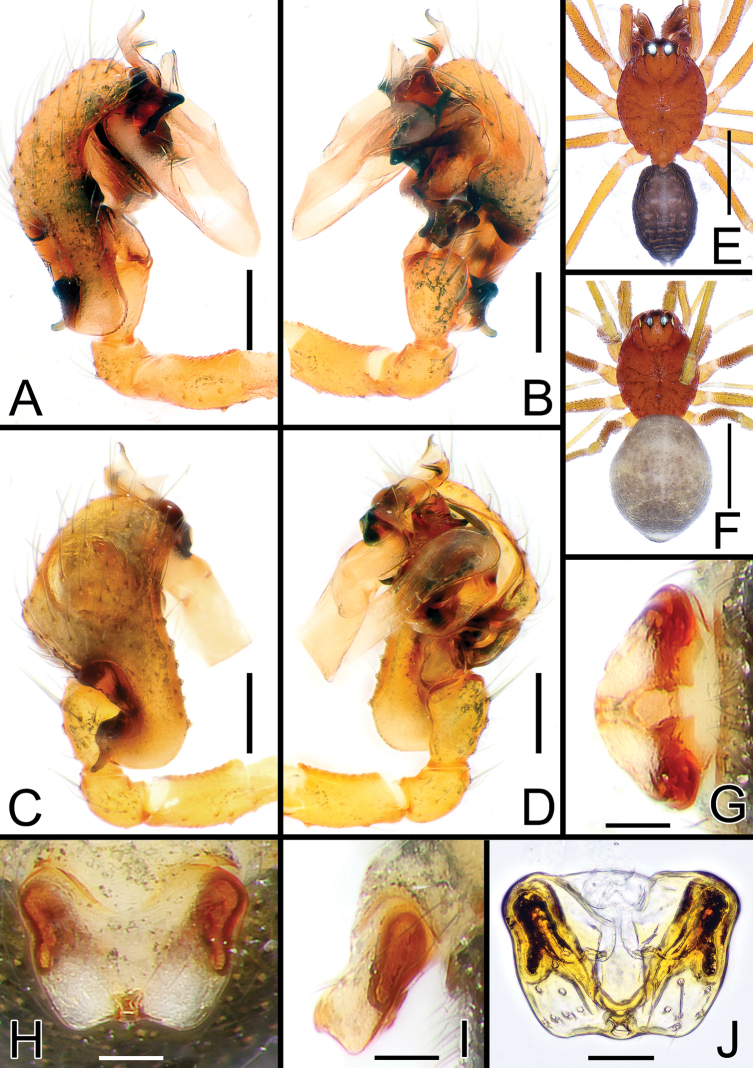
*Solenysalongqiensis***A** male palp, prolateral **B** ditto, retrolateral **C** ditto, dorsal **D** ditto, ventral **E** habitus of male, dorsal **F** habitus of female, dorsal **G** epigyne, posterior **H** ditto, ventral **I** ditto, lateral **J** vulva, dorsal. Scale bars: 0.05 mm (**A–D, G–J**); 0.5 mm (**E, F**).

Habitus of male as in Fig. 9E, habitus of female as in Fig. 9F. Palp as in Fig. 9A−D, embolic division as in Fig. 11G, H; epigyne and vulva as in Fig. 9G−J. See Tu and Li (2006) for a detailed description.

##### Remarks.

This species was originally described from mainland China by Tu and Li (2006) and its presence in Taiwan was later confirmed by Tanasevitch (2011). Tian et al. (2022) in their map on *Solenysa* species distribution, report several records of the *S.longqiensis* group in Taiwan without specifying which species in particular they refer to. Our data from central Taiwan confirm that *S.longqiensis* is widespread across the island. Additionally, we report it as sympatric with the Taiwanese endemic and closely related *S.yangmingshana*, as we collected the two species together in the same habitat in more than one location (Fig. 13).

#### ﻿ Solenysa yangmingshana

Taxon classificationAnimaliaAraneaeLinyphiidae

Tu, 2011

A9C73F39-9FF0-5CCB-8992-2AABB51141D0

Figs 10A−J , 11I, J

Solenysa yangmingshana Tu in Tu & Hormiga, 2011: 503, fig. 11A–G (♂♀).

##### Material examined.

**Taiwan: Nantou County**, Ren’ai, • 2 ♀, Huisun Forest Area, 740 m, 24.08967°N, 121.03529°E, broadleaf forest litter along the trail, 12.July.2023 F. Ballarin leg. (FBPC) • 2 ♂, same locality, 930 m, 24.08421°N, 121.03438°E, rather humid broadleaf forest litter on a gentle slope, 14.July.2023, F. Ballarin leg. (FBPC) • 4 ♀, Menggu Waterfall, 910 m, 24.02824°N, 121.08067°E, broadleaf forest litter along the trail, 17.July.2023, F. Ballarin leg (NSMT) • **Taichung City Metropolitan Area**, 1 ♀, Dongshi District, Daxue Mountain, 860 m, 24.21595°N, 120.88943°E, broadleaf forest leaf litter along the trail, 18.July.2023, F. Ballarin leg. (TARI).

##### Type locality:

Mt. Yangmingshan, Taipei City, Taiwan (~ 25.171°N, 121.553°E).

##### Diagnosis.

See Tu and Hormiga (2011).

##### Description.

**Figure 10.**
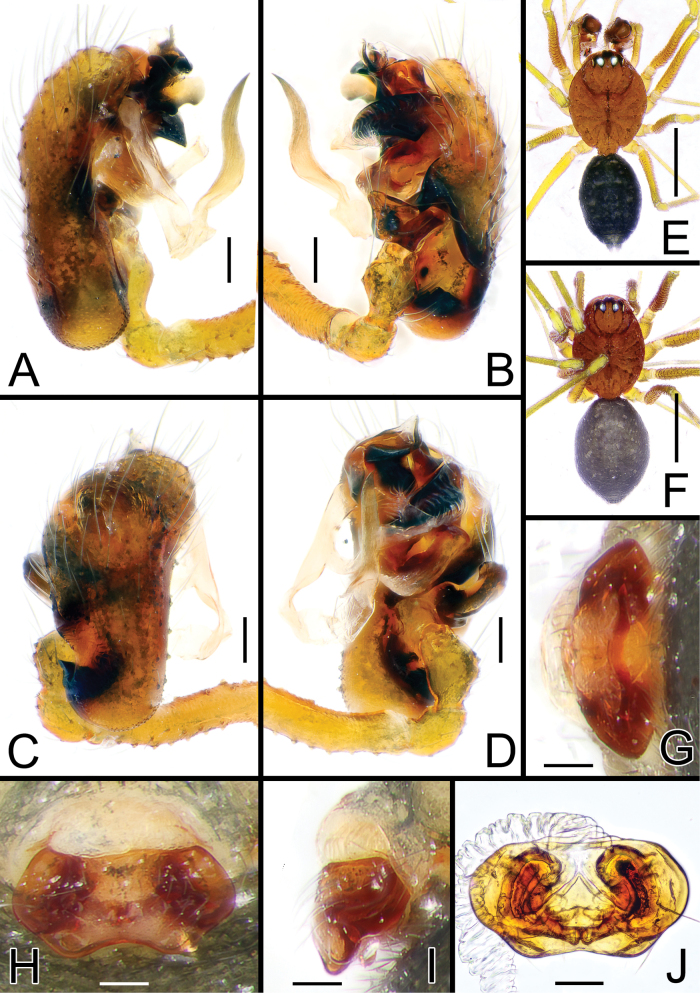
*Solenysayangmingshana***A** male palp, prolateral **B** ditto, retrolateral **C** ditto, dorsal **D** ditto, ventral **E** habitus of male, dorsal **F** habitus of female, dorsal **G** epigyne, posterior **H** ditto, ventral **I** ditto, lateral **J** vulva, dorsal. Scale bars: 0.05 mm (**A–D, G–J**); 0.5 mm (**E, F**).

**Figure 11.**
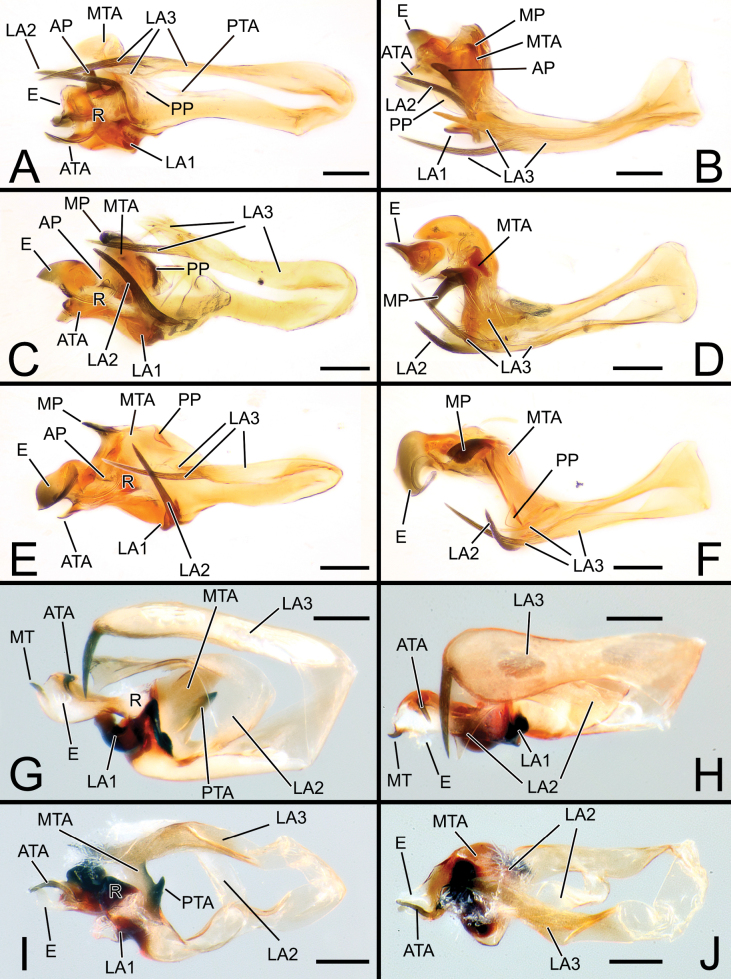
Embolic divisions of *Solenysa* species **A***S.reflexilis* from Yakushima, ventral **B** ditto, retrolateral **C***S.macrodonta* from Hiroshima, ventral **D** ditto, retrolateral **E***S.trunciformis* from Okayama, ventral **F** ditto, retrolateral **G***S.longqiensis*, ventral **H** ditto, retrolateral **I***S.yangmingshana***J** ditto, retrolateral. (N.B., in **A−D** embolus is broken). Abbreviations: AP anterior protrusion of MTA; ATA anterior terminal apophysis; E embolus; LA1 anterior branch of lamella; LA2 median branch of lamella; LA3 posterior branch of lamella; MP median protrusion of MTA; MT median tooth on anterior terminal apophysis; MTA median terminal apophysis; PP posterior protrusion of MTA; PTA posterior terminal apophysis; R radix. Scale bars: 0.05 mm.

Habitus of male as in Fig. 10E. Total length: 1.56. Prosoma 0.85 long; 0.60 wide. Clypeus 0.38 long. Cephalic area distinctly elevated in both sexes. Carapace oval, lacking lateral lobes. Carapace, chelicera, mouth parts, and sternum uniformly brick-red. Chelicera with four promarginal and three retromarginal teeth. Legs uniformly red-yellowish. TmI = 0.43. TmIV absent. Length of legs as follows: I 3.71 (1.01, 0.20, 1.04, 0.86, 0.60), II 3.21 (0.88, 0.18, 0.84, 0.75, 0.56), III 2.56 (0.78, 0.18, 0.60, 0.56, 0.44), IV 3.19 (0.92, 0.17, 0.83, 0.73, 0.54). Opisthosoma uniformly dark grey with some faint lighter dots on dorsal side. Some individuals with one light mark on dorsal-posterior part of opisthosoma. Palp as in Fig. 10A−D, embolic division as in Fig. 11I, J.

Habitus of female as in Fig. 10F. Total length: 1.55. Prosoma 0.79 long; 0.53 wide. Clypeus 0.28 long. Length of legs as follows: I 3.30 (0.95, 0.17, 0.89, 0.74, 0.55), II 2.91 (0.85, 0.16, 0.77, 0.62, 0.51), III 2.37 (0.68, 0.16, 0.55, 0.52, 0.46), IV 2.92 (0.89, 0.17, 0.75, 0.63, 0.48). Color and other features as in male. Epigyne and vulva as in Fig. 10G−J.

See Tu and Hormiga (2011) for a detailed description of genitalia.

##### Remarks.

Species endemic to the island of Taiwan (Fig. 13). As far as we know, this species was previously recorded only from a few specimens collected in the north of the Island. Similarly to the previously mentioned *S.longqiensis*, *S.yangmingshana* is widespread across Taiwan and both species share the same habitat. In the original description by Tu and Hormiga (2011), the coordinates of the type locality are incorrectly reported and refer to the type locality of *S.longqiensis*. Additionally, in their distribution map, Tu and Hormiga (2011: 517) erroneously report the type locality as being in central Taiwan, while it is located in the north of the island. They also mention that the original male holotype was dried out and could not be properly measured thus limiting their description to the genitalia. Accordingly, here we redescribe the habitus of both male and female of this species reporting the related measurements.

#### ﻿ Solenysa lanyuensis

Taxon classificationAnimaliaAraneaeLinyphiidae

Tu, 2011

CD05F250-E884-55E2-BDC2-D37DFDB6C1A2

Solenysa protrudens Tu in Tu and Li, 2006: 94, figs 31−39 (♂♀, misidentified).
S. lanyuensis : Tu and Hormiga 2011: 515, fig. 7C (♂♀).

##### Type locality.

Lanyu Island (= Orchid Island), Taitung County, Taiwan (see remarks)

##### Diagnosis.

See Tu and Li (2006)

##### Description.

See Tu and Li (2006)

##### Remarks.

The type series of *S.lanyuensis* (3 ♂ and 2 ♀) was originally misidentified as *S.protrudens* by Tu and Li (2006). Later, *S.lanyuensis* was recognized as a new species and described based on the same specimens by Tu and Hormiga (2011). The coordinates of the type locality reported by both Tu and Li (2006) and Tu and Hormiga (2011) refer to a coastal area in southeast Taiwan and are likely incorrect since the name of the locality, from which the species also takes its name, refer to Lanyu, a small volcanic island located ca. 75 km far from the Taiwanese coast. The exact collecting location of the type series on the island remains unknown. In 2019, we had the opportunity to conduct an extensive survey on Lanyu Island, which led to the discovery of several rare litter-dwelling spiders, including linyphiids (Ballarin et al. 2021). However, despite our extensive collecting effort, we failed to find any specimens of *Solenysa* on the island. As far as we know, this species remains recorded only from two localities, Lanyu Island from the original description and the location in central Taiwan reported by Tian et al. (2022, reported in the map but locality not specified). Additional collection in southwest Taiwan may help to clarify the distribution of this species.

###### ﻿Other comparative material examined

***Solenysamellotteei* Simon, 1894: Japan: Tokyo Pref.**, 1 ♂, 1 ♀, Hachioji City, Naganumamachi, Naganuma Park (長沼公園), 150 m, 35.637°N, 139.368°E, forest leaf litter on a slope, 07.Aug.2019, F. Ballarin leg. (FBPC).

***Solenysaogatai* Ono, 2011: Japan: Shizuoka Pref.**, 1 ♂, Fujinomiya City, Nukudo, Hoshiyama, Myojoyama Park (明星山公園), 17.Nov.2014, A. Tanikawa leg. (FBPC) • **Kanagawa Pref.**, 1 ♂, 1 ♀, Ashigarashimo-gun, Manazaru, Manatsuru cape, 90 m, 35.14409°N, 139.15556°E, thick leaf litter in a primary forest, 28.Jul.2021, F. Ballarin leg. (FBPC).

### ﻿Molecular analysis

**Figure 12.**
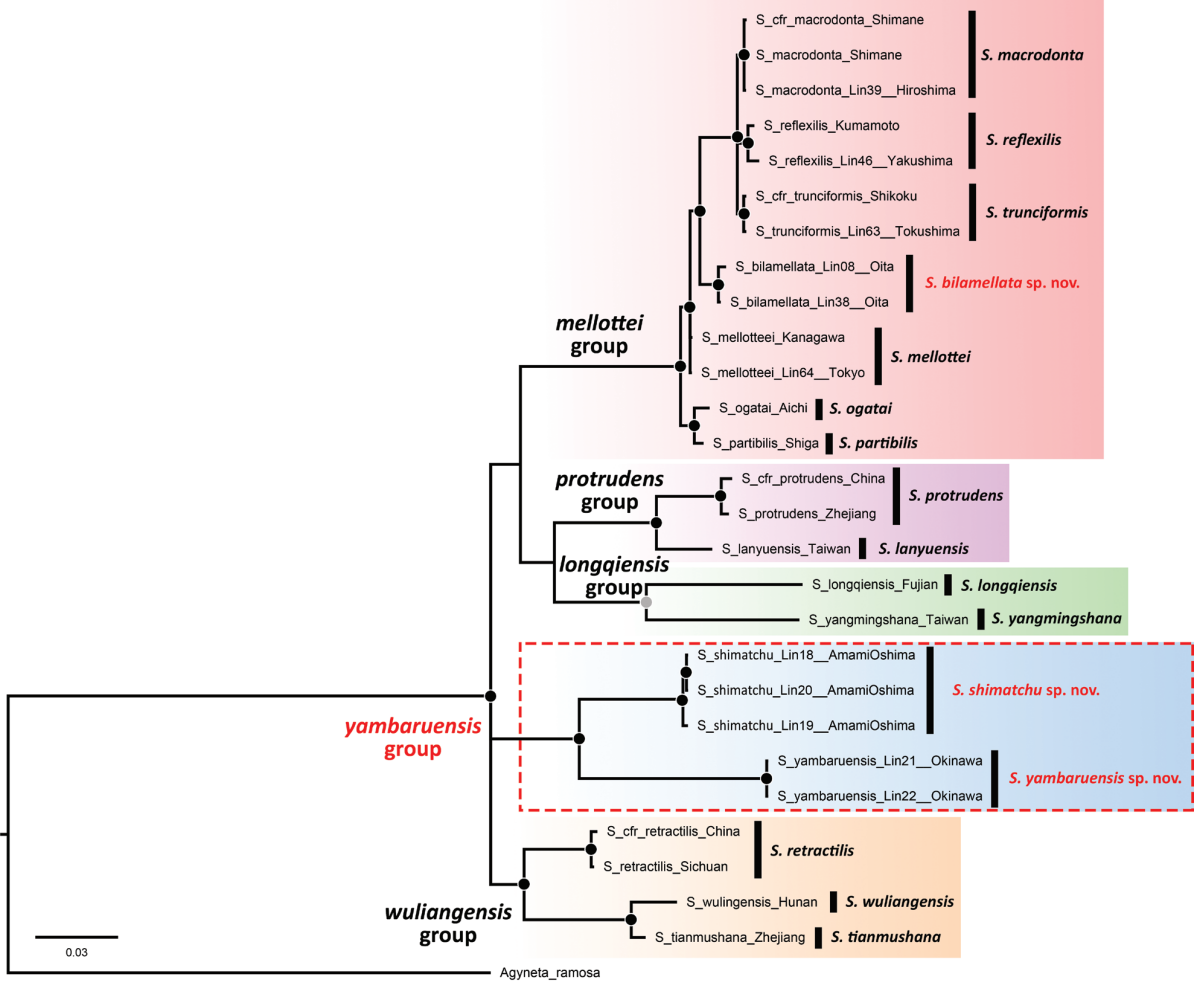
Phylogenetic tree based on combined COI and H3 gene fragments reconstructed using maximum likelihood (ML) on RAxML and Bayesian inference (BI) on MrBayes. Support at each node denotes the ML bootstrap value (BV) and BI posterior probability (PP). Nodes highly supported by at least one method (BV ≥ 75 or PP ≥ 0.95) are highlighted by a black dot, nodes with medium support (BV ≥ 70 or PP ≥ 0.90) are reported in grey, low supported nodes lack a dot. Branch lengths were scaled to the number of substitutions per site. *Solenysa* species groups are highlighted with different colors. New taxa discussed in this work are reported in red.

The final dataset used for the phylogenetic analysis included 27 ingroup terminals and 16 *Solenysa* species. The concatenated sequences had a total of 1047 bp (COI = 696 bp, H3 = 351 bp). Both the ML and BI analyses produced the same tree topology, with similar support for the wide majority of the nodes. The resulting tree is shown in Fig. 12.

**Figure 13.**
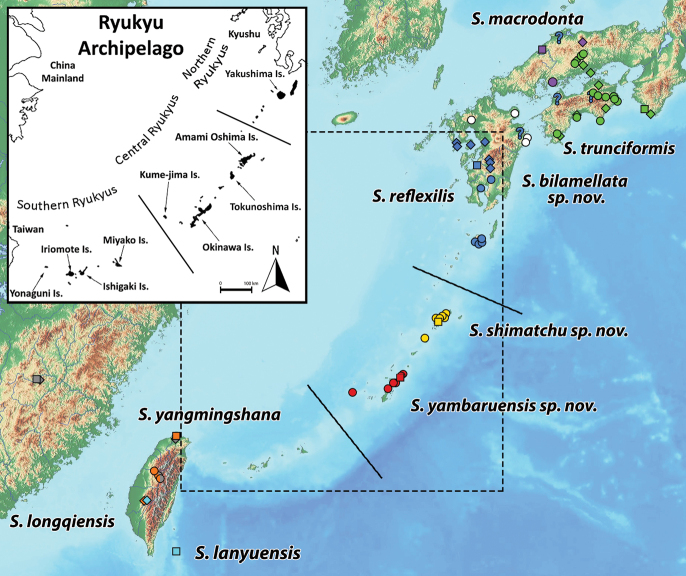
Distribution of *Solenysa* species in the Ryukyu Archipelago and surrounding areas. The Ryukyu Archipelago is marked with a dashed line. Diamonds refer to records of *Solenysa* spp. from the literature (Shinkai et al. 2024; WSC 2024), dots refer to new records, squares indicate the species type locality, and question marks indicate uncertain records of *S.reflexilis*.

In the tree, the separation of the *Solenysa* species identified by morphology is endorsed by strongly supported nodes. The new species from Kyushu, *S.bilamellata* sp. nov., is reconstructed with strong support as monophyletic and closely related to the other species of the *S.mellotteei* group distributed in mainland Japan. The two new species from the Ryukyus, *S.shimatchu* sp. nov. and *S.yambaruensis* sp. nov., are both strongly supported. Together they form a distinct monophyletic lineage with a long basal branch and well separated from all the other known species groups. This further supports the establishment of a new species group, the *S.yambaruensis* group, to accommodate them. All four previously known *Solenysa* species groups, although generally poorly supported at the basal node, are reconstructed as distinct lineages, separated to each other by long basal branches. These results are consistent with the morphological analysis and previous studies.

The interspecific genetic diversity of *Solenysa* species is reported in Table 2, ranging from 0.4% to 13.5%, with an average of 9%. Genetic diversity between species groups ranges from 7.7% to 13.5% (average 10.6%). The average genetic diversity within the species groups is as follows: *S.yambaruensis* group = 11%, *S.longqiensis* group = 9.7%, *S.wulingensis* group = 5.3%, *S.protrudens* group = 4.2%, *S.mellotteei* group = 2.6%. The *S.mellotteei* group shows the lowest interspecific ranging spanning from 0.4% to 4.4%. *Solenysabilamellata* sp. nov. shares a genetic distance with other species of the *S.mellotteei* group ranging from 1.2% to 4.4% (average 3%). The lowest genetic distance in the group is calculated between *S.mellotteei* and *S.partibilis*, revealing a surprisingly low value of only 0.4%. Fresh samples of *S.partibilis* were not available for our pairwise analysis, thus for this species we relied on sequences harvested from GenBank. Given the well-known problem of misidentifications in online genetic databases, we cannot exclude that the COI barcode attributed to *S.partibilis* and used by us for the pairwise analysis may actually belong to another misidentified species in the *S.mellotteei* group.

**Table 2.** Uncorrected Pairwise-distance between the species and species groups of *Solenysa* based on the barcode COI partial sequences.

Species		*S.bilamellata* sp. nov.	*S.lanyuensis*	*S.longqiensis*	*S.macrodonta*	*S.mellotteei*	*S.ogatai*	*S.partibilis*	*S.protrudens*	*S.reflexilis*	*S.retractilis*	*S.tianmushana*	*S.shimatchu*	*S.trunciformis*	*S.wulingensis*	*S.yambaruensis*
	**Species group**	mellotteei gr.	protrudens gr.	longqiensis gr.	mellotteei gr.	mellotteei gr.	mellotteei gr.	mellotteei gr.	protrudens gr.	mellotteei gr.	wulingensis gr.	wulingensis gr.	yambaruensis gr.	mellotteei gr.	wulingensis gr.	yambaruensis gr.
***S.bilamellata* sp. nov.**	mellotteei gr.															
***S .lanyuensis***	protrudens gr.	0.108														
***S.longqiensis***	longqiensis gr.	0.115	0.088													
***S.macrodonta***	mellotteei gr.	0.029	0.104	0.108												
***S.mellotteei***	mellotteei gr.	0.015	0.108	0.110	0.026											
***S.ogatai***	mellotteei gr.	0.031	0.115	0.117	0.040	0.009										
***S.partibilis***	mellotteei gr.	0.022	0.108	0.110	0.035	0.004	0.013									
***S.protrudens***	protrudens gr.	0.115	0.042	0.088	0.102	0.106	0.113	0.106								
***S.reflexilis***	mellotteei gr.	0.034	0.104	0.104	0.013	0.029	0.044	0.035	0.102							
***S.retractilis***	wulingensis gr.	0.102	0.077	0.086	0.099	0.097	0.104	0.097	0.084	0.095						
***S.tianmushana***	wulingensis gr.	0.097	0.079	0.095	0.095	0.097	0.104	0.097	0.088	0.091	0.060					
***S.shimatchu***	yambaruensis gr.	0.112	0.095	0.117	0.111	0.115	0.115	0.108	0.102	0.113	0.097	0.097				
***S.trunciformis***	mellotteei gr.	0.035	0.102	0.106	0.007	0.029	0.038	0.033	0.099	0.015	0.095	0.091	0.110			
***S.wulingensis***	wulingensis gr.	0.108	0.093	0.097	0.095	0.104	0.106	0.108	0.095	0.095	0.071	0.029	0.097	0.091		
***S.yambaruensis***	yambaruensis gr.	0.114	0.132	0.130	0.120	0.118	0.132	0.130	0.135	0.117	0.113	0.117	0.109	0.128	0.121	
***S.yangmingshana***	longqiensis gr.	0.117	0.093	0.097	0.110	0.115	0.121	0.115	0.115	0.106	0.102	0.106	0.113	0.106	0.106	0.130

## ﻿Discussion and conclusions

Despite being a relatively well-studied linyphiid genus, the diversity of *Solenysa* still seems far to be completely defined. Our study increases the number of species from Japan from six to nine, and the total number of known *Solenysa* species from 15 to 18. The *S.mellotteei* group is confirmed as the most speciose within the genus, consisting solely of species distributed in mainland Japan. Nevertheless, this group also exhibits the lowest interspecific diversity among the species groups, suggesting a recent diversification, as previously proposed by other studies (Tian et al. 2022). The discovery of *Solenysabilamellata* sp. nov. from Kyushu, along with the morphological differences observed among allopatric populations in other species of the same group, suggests that the true magnitude of the diversity of the *S.mellotteei* group may still be underestimated and that additional species may remain undetected in Japan.

Recent taxonomic studies on spiders in the Ryukyus have highlighted high levels of heterogeny and endemism at small geographic scales, proposing the role of the archipelago as a hotspot of spider diversity (Xu et al. 2019; Ihara et al. 2021; Suzuki et al. 2022; Ballarin and Eguchi 2022, 2023). The discovery of two undescribed *Solenysa* species in the Ryukyus, belonging to a new and distinct species group, further supports this hypothesis and fills a gap that was present in the distribution of the genus. However, the position of this species group within the genus remains poorly defined in our phylogenetic study and requires further comprehensive analyses. Linyphiidae are well-known to perform ballooning (Duffey 1998) and thus allowing them to potentially disperse in distant lands and islands. Yet, our study supports a strong level of endemism in *Solenysa* in both the Ryukyus and mainland Japan, possibly linked to the specific microhabitat conditions needed by these spiders. This trait, combined with the genus’ putative old origin and high local diversification in mainland Japan, suggest that *Solenysa* may represent a valuable model subject for future biogeographic studies. Further research on this genus my offer new opportunities to shed light on the origin, adaptive radiation, and potential ancient routes of colonization of terrestrial arthropods in the Ryukyus and mainland Japan.

## Supplementary Material

XML Treatment for Solenysa

XML Treatment for Solenysa shimatchu

XML Treatment for Solenysa yambaruensis

XML Treatment for Solenysa bilamellata

XML Treatment for Solenysa reflexilis

XML Treatment for Solenysa macrodonta

XML Treatment for Solenysa trunciformis

XML Treatment for Solenysa longqiensis

XML Treatment for Solenysa yangmingshana

XML Treatment for Solenysa lanyuensis

## ﻿Additional information

### Author ORCIDs

Francesco Ballarin https://orcid.org/0000-0003-1417-2519

Katsuyuki Eguchi https://orcid.org/0000-0002-1054-1295

### Data availability

All of the data that support the findings of this study are available in the main text.
